# Fluorescent NHC Gold(I) Complexes as Tools for Target Identification and Metallomic Studies in Cancer Cells

**DOI:** 10.1021/acs.inorgchem.6c01796

**Published:** 2026-08-03

**Authors:** Ester Giorgi, Matteo Boldrini, Francesca Binacchi, Damiano Cirri, Michele Mannelli, Tania Gamberi, Massimo Guelfi, Matteo Becatti, Lara Massai, Chiara Gabbiani, Alessandro Pratesi, Luigi Messori

**Affiliations:** † Department of Chemistry and Industrial Chemistry, University of Pisa, Via G. Moruzzi 13, 56124 Pisa, Italy; ‡ Department of Experimental and Clinical Biomedical Sciences “Mario Serio”, 9300 University of Florence, Viale G.B. Morgagni 50, 50134 Firenze, Italy; § Department of Chemistry “U. Schiff”, University of Florence, Via della Lastruccia 3-13, 50019 Sesto Fiorentino, Italy

## Abstract

In recent years, gold­(I) complexes have attracted increasing interest as an alternative to conventional metallo-chemotherapeutic agents. Their mode of action differs significantly from that of traditional platinum-based drugs. However, the identification of their intracellular targets and the elucidation of their mechanisms of action remain major challenges, largely due to the lack of suitable molecular tools for tracking these compounds in biological systems. To address these issues, three novel gold­(I) complexes bearing two different fluorescent labels were synthesized and characterized. These complexes include an *N*-heterocyclic monocarbene gold­(I) complex **3** and the corresponding biscarbene **4**, both of which are functionalized with an anthracenyl moiety, as well as an NHC gold­(I) monocarbene complex bearing a BODIPY label **5**. The specific aim of this study was to generate trackable metallodrug models for mechanistic and metallomics-oriented research. These compounds were characterized using ^1^H and ^13^C NMR spectroscopy and elemental analysis. The crystallographic structure of the anthracenyl gold monocarbene was also determined. Interaction studies with human serum albumin (HSA) using UV–vis absorption spectroscopy and ESI-MS provided clear evidence of metallodrug–protein interactions. The uptake of the complexes in A2780 ovarian cancer cells was measured using fluorescence-activated cell sorting (FACS) cytofluorimetry, which indicated strong cellular uptake for the anthracenyl-labeled compounds **3** and **4**. A cell fractionation approach was used for the quantitative analysis of subcellular drug disposition. These approaches collectively enabled a direct visualization of the intracellular fate of the gold complexes, providing a framework for correlating cellular distribution with biological activity. Finally, the gold complexes bearing the anthracenyl probe were evaluated for their cytotoxic activity in cisplatin-sensitive and -resistant A2780 cancer cells; the BODIPY complex could not be evaluated due to its insolubility. Overall, this study provides solid evidence that gold complexes bearing the anthracenyl probe retain significant cytotoxic properties and enable monitoring the uptake and fate of these gold species inside cancer cells, thus providing a proof-of-concept that fluorescently labeled gold complexes can serve as powerful tools for mechanistic investigations, including metallomics and proteomics-oriented studies aimed at identifying molecular targets and affected pathways.

## Graphical abstract

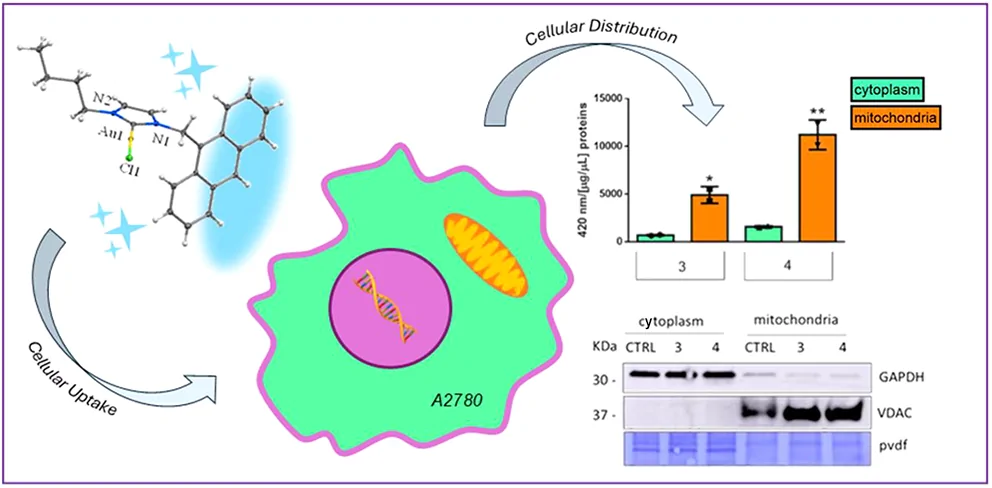

## 1 Introduction

Since Lorber et al. discovered the antiproliferative activity of auranofin (AF) on HeLa cervical cancer cells in 1979, gold complexes have attracted increasing attention as potential chemotherapeutic agents.^1–4^ This is partly because gold-based drugs have different pharmacological profiles to platinum-based ones, suggesting new biological mechanisms that could overcome resistance to cisplatin in tumors. It is believed that gold compounds exert their biological activity by targeting the cysteine and selenocysteine side chains in the active site of the key enzyme thioredoxin reductase. This results in significant disruption to cell redox homeostasis and ultimately induces apoptosis.^5–9^ However, this mechanistic picture is still incomplete, and increasing evidence suggests that gold complexes interact with multiple cellular targets and pathways beyond thioredoxin reductase.

Over the years, numerous gold complexes have been synthesized and evaluated for their cytotoxic properties on various cancer cell lines.^10^ *N*-heterocyclic carbene (NHC) compounds, in particular, have emerged as highly promising within the broad family of gold­(I) complexes, thanks to their high chemical stability, strong metal–ligand bonds, and reduced susceptibility to ligand exchange with biomolecules such as serum proteins.^11,12^ The ease with which NHCs can be synthetically modified to adjust lipophilicity and charge- and consequently their biological behavior- coupled with these characteristics, has enabled the design of compounds with promising cytotoxic activities and a variable degree of selectivity toward cancer cells.^13–15^ Despite these advances, the detailed biological mechanisms and specific molecular targets of gold complexes remain unclear. A major limitation in this context is the lack of experimental strategies that allow simultaneous tracking of metallodrug distribution and identification of their interacting biomolecular partners within the cellular environment.

To design new drug candidates or improve existing ones, it is crucial to have an in-depth understanding of the mechanistic aspects of metal complexes from a Systems Biology standpoint. Integrating molecular imaging tools, such as fluorescent tagging, enables the real-time tracking of gold complexes within cells. This provides valuable insights into their pharmacokinetics, subcellular localization and engagement with targets. In particular, the incorporation of fluorescent probes into metallodrug scaffolds represents a promising chemical biology strategy to transform active compounds into traceable probes, enabling their use in uptake studies, subcellular localization, and downstream target identification workflows.

Accordingly, the present study involved the synthesis and characterization of a small group of Au­(I) fluorescent complexes (Figure 1), with the aim of exploiting their fluorescence properties in uptake and biodistribution studies via flow cytometry and conventional cell fractionation methods. This could help to identify potential targets for these complexes and establish links to altered cellular pathways. Importantly, this approach was conceived not only to evaluate pharmacokinetic properties but also to establish a platform for future metallomics and proteomics investigations aimed at elucidating the molecular mechanisms of action of these compounds.

**Figure 1:**
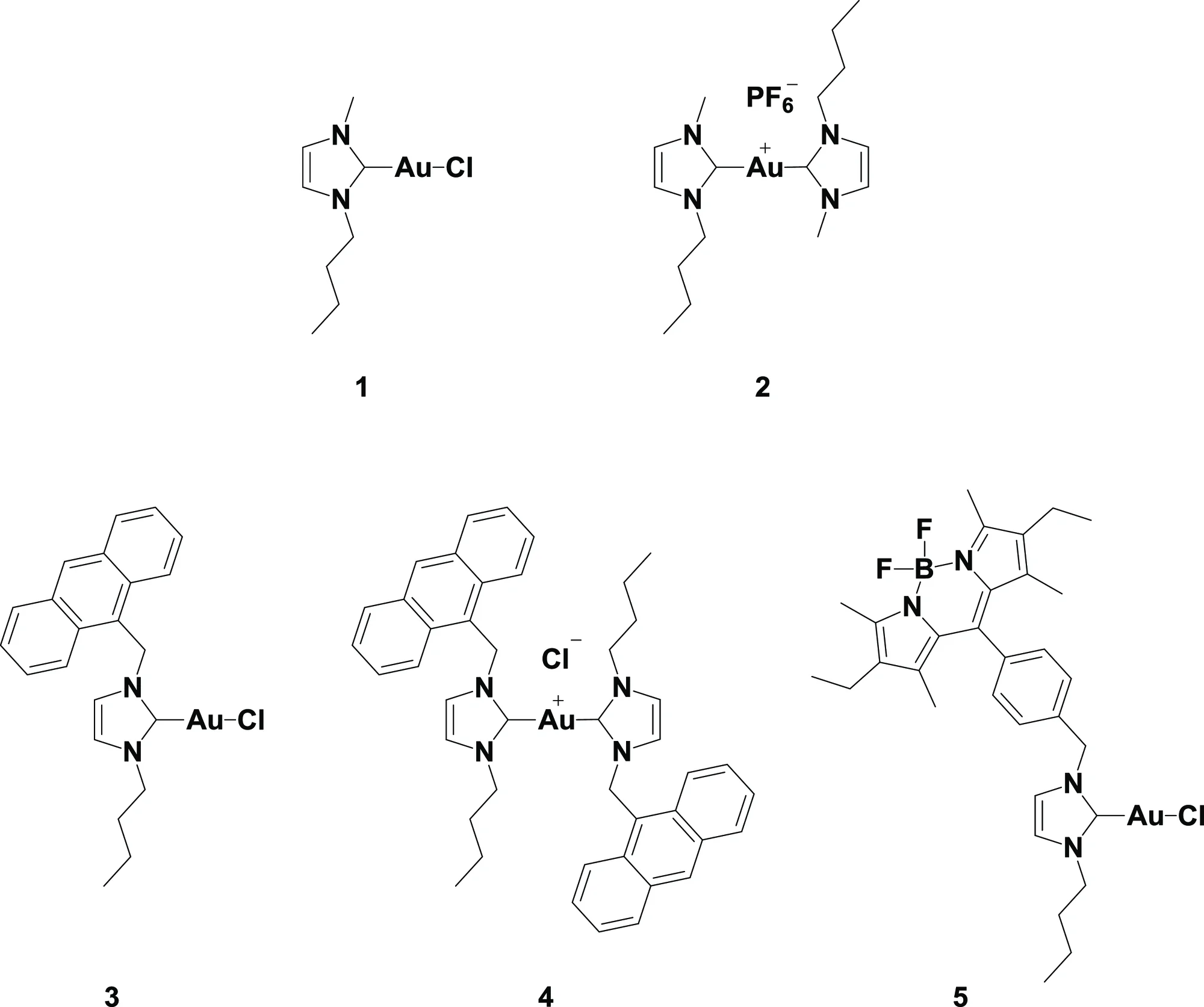
Reference complexes (**1**, **2**) and the panel of fluorescently labeled complexes synthesized for this work (**3**–**5**).

These novel complexes were designed by modifying two previously synthesized complexes: (1-butyl-3-methylimidazole-2-ylidene) gold­(I) (**1**) and bis­(1-butyl-3-methylimidazole-2-ylidene) gold­(I) (**2**) (Figure 1).^16^ The use of well-characterized parent scaffolds allowed us to introduce fluorescent handles into Au­(I)–NHC frameworks with established biological activity. This design was intended to generate trackable metallodrug models while retaining relevant cytotoxic properties. However, the introduction of extended aromatic fluorophores, such as anthracene, may alter lipophilicity, biomolecular recognition, and cellular behavior; therefore, the biological properties of the labeled compounds were experimentally reassessed and compared with those of the parent complexes.

Hence, the methyl substituent of the parent carbenes was replaced with two different fluorescent probes: 9-methylanthracene and 4,4-difluoro-8-(4-chloromethylphenyl)-1,3,5,7-tetramethyl-2,6-diethyl-4-bora-3a,4a-diaza-s-indacene (BODIPY). Anthracene and BODIPY were selected as fluorescent tags because of their distinct emission properties, which make them potentially suitable for fluorescence-based cellular tracking. Anthracene derivatives typically display blue emission, whereas BODIPY derivatives generally emit in the green spectral region.^17^ In the present study, the anthracenyl-labeled Au­(I)–NHC compounds proved suitable for FACS-based uptake and subcellular distribution studies, while the BODIPY-labeled compound displayed a broader fluorescence response across different detection channels but poorer aqueous compatibility.

## 2 Results and Discussion

### 2.1 Synthesis and Characterization

The complexes studied (**3–5**) were synthesized following Scheme 1. All the resulting complexes were characterized using ^1^H, ^13^C NMR and elemental analysis; the ^19^F NMR spectrum was also recorded for complex **5** (see SI). The BODIPY probe (**10**) was prepared by adapting the procedure reported in the literature.^17^ 3-ethyl-2,4-dimethylpyrrole (**8**) was reacted with 4-(chloromethyl)­benzoyl chloride (**7**) under inert atmosphere. The intermediate thereby obtained (**9**) was then treated with an excess of NEt_3_ and BF_3_·OEt_2_. The addition of these two reagents, made drop by drop, caused the formation of a white fog. The red solution was washed with a 10% HCl aqueous solution to protonate nitrogenous byproducts and extract them from the mixture. The organic phases were then collected and purified using silica gel flash column chromatography (eluent: CH_2_Cl_2_/hexane 1:1), the compound of interest was the second species eluted from the column. The yellow fractions were collected and solvent removal under vacuum led to the formation of a red solid. The product was obtained in a low yield (16%). The synthesis of the anthracenyl imidazolium ligand (**13**) was carried out by reacting 1-butylimidazole (**11**) and 9-(chloromethyl)­anthracene (**12**) in 1:1 stoichiometric ratio and using acetonitrile as solvent. A yellow solution was obtained. The reaction was kept at reflux for 48 h, then the solvent was removed under reduced pressure. The recovered solid residue was washed with diethyl ether to eliminate unreacted imidazole. The final product was obtained as a yellow-pale solid in good yield (85%). The BODIPY imidazolium salt (**14**) was obtained by dissolving **10** in an excess of **11** and kept under stirring for 24 h. Diethyl ether was then added to precipitate the product and dissolve the unreacted imidazole. Compound **14** was obtained as a red solid with a 68% yield. The ^1^H NMR spectra comparison of the product and the reagent (Figure S7a) shows a shift of the CH_2_ signal from 4.66 to 5.79 ppm, caused by the binding to the positive nitrogen, which indicates the formation of the product. The gold monocarbenes (**3**, **5**) were obtained by transmetalation of the respective silver compounds (**15**, **16**). **13** and **14** were treated with a slight excess of Ag_2_O in anhydrous CH_2_Cl_2_. The mixture was kept under stirring in the dark for 20 h. The formation of the silver carbene was assessed by the disappearance of the imidazole hydrogen signal at 11.19 ppm in the ^1^H NMR spectrum (Figure S7b). The mixture was filtered over Celite to eliminate unreacted Ag_2_O. One equivalent of [AuCl­(SMe_2_)] was added to the solution, which was kept under stirring in the dark for 4 h. The mixture became cloudy due to the precipitation of AgCl and partial reduction of gold. The mixture was filtered again, leaving a purple solid on the Celite. Complex **3** was recovered by adding pentane, obtaining the product as a red solid (yield: 65%). Complex **3** was also recrystallized by layering hexane onto a solution of the product in CH_2_Cl_2_. The resulting crystals were subsequently analyzed through X-ray diffraction (see Supporting Information for crystallographic data), confirming the linear geometry of the complex (Figure 2).

**Figure 2:**
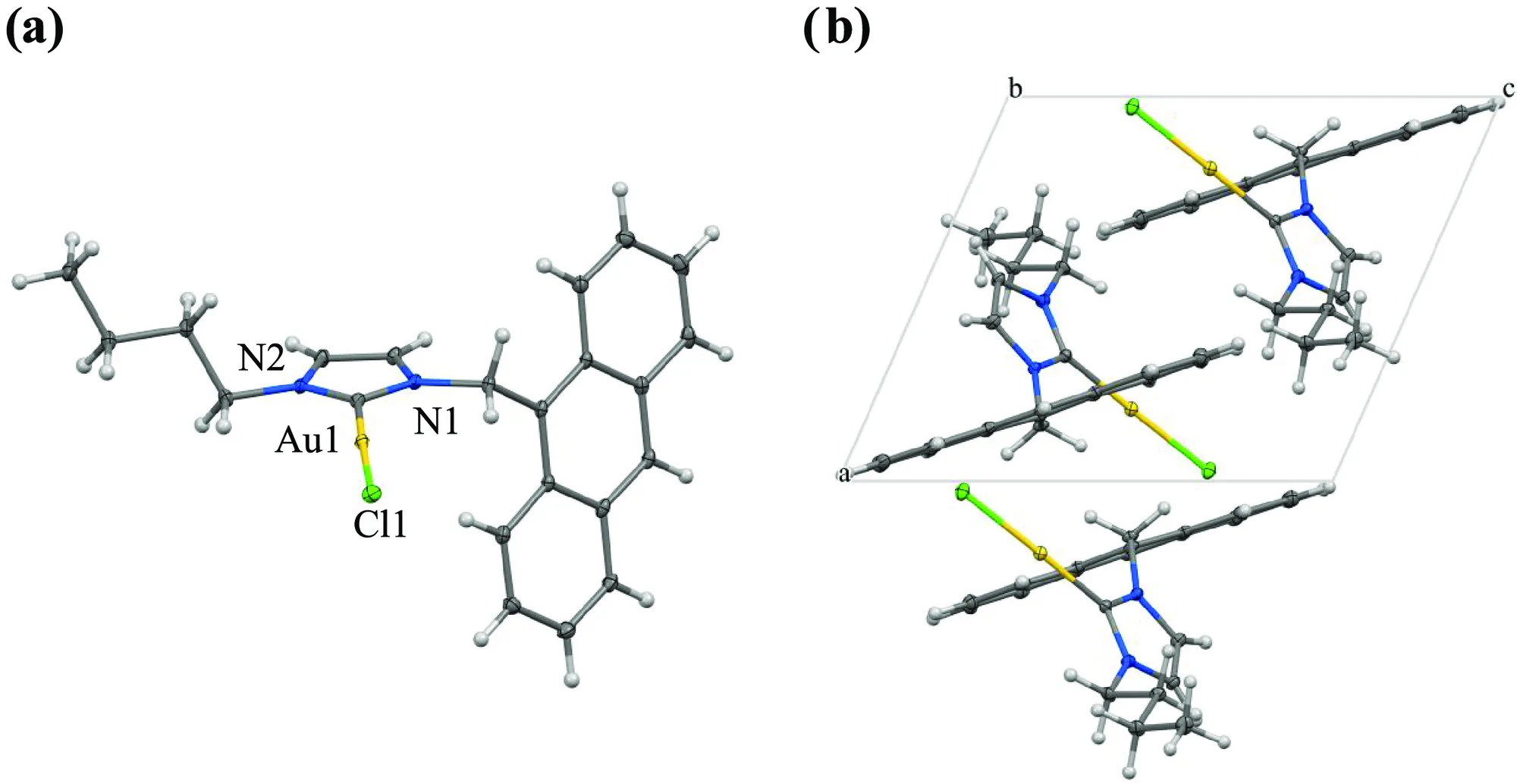
(a) Molecular structure of complex (**3**). (b) Crystal packing viewed along the ac plane, highlighting how the extended π-system of the anthracenyl fragment of the N-heterocyclic carbene ligand engages in two distinct types of π-interactions: one between units within the unit cell and another with units outside the cell. Au in yellow, Cl in green and N in blue, ellipsoids are drawn at the 30% probability level; all hydrogen atoms are shown as spheres with a fixed radius of 0.15 Å.

**Scheme 1:**
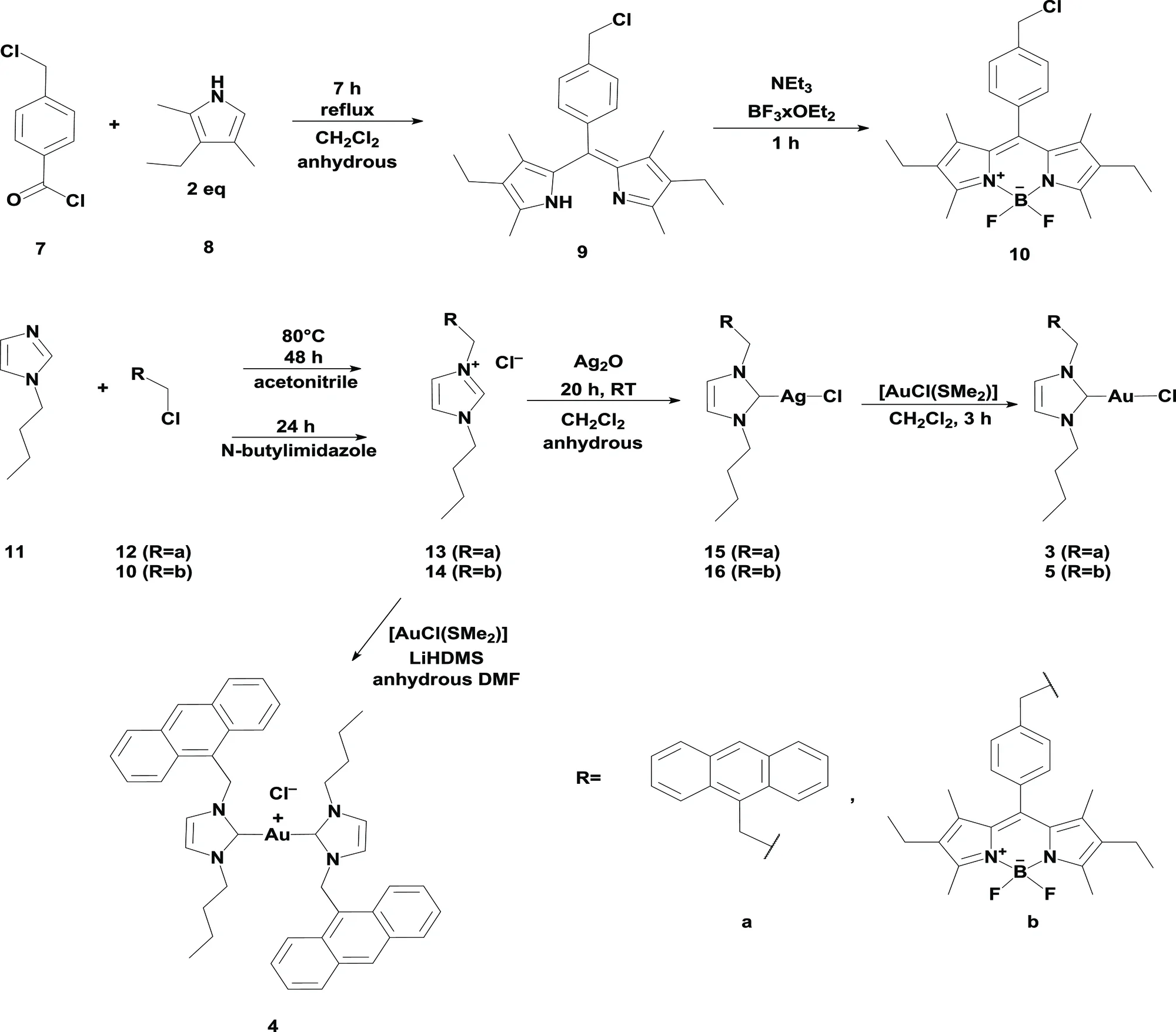
Synthesis of Complexes **3**–**5**

The most significant geometric parameters are summarized in Tables S1–S3. The gold center adopts a pseudolinear geometry, with a C–Au–Cl bond angle of 177.09°. The Au–C_carb_ (1.979 Å) and Au–Cl (2.2883 Å) bond lengths are consistent with previously reported values for [AuCl­(NHC)]-type complexes.^18–21^ The anthracenyl fragment is oriented almost perpendicular to the plane of the N-heteroaromatic carbene, forming an interplanar angle of 77.24° (see Figure S8). Within the crystal lattice, complex (**3**) displays an Au···Au distance of 3.769 Å between the closest units (Figure S9), while in other directions, these distances increase in range between 5.700 and 10.659 Å, indicating a rather modest aurophilic character compared to other Au­(I) complexes.^19^ The anthracene moieties of two units within the unit cell exhibit a plane-to-centroid distance of 3.351 Å and a centroid-to-centroid distance of 7.988 Å, suggesting significant offset stacking between the aromatic rings. In contrast, when considering a symmetry-related unit outside the unit cell, the interplanar distance increases slightly to 3.421 Å, while the centroid-to-centroid distance decreases significantly to 3.816 Å, indicating a relative alignment favorable for noncovalent interactions (Figure S10). To better investigate this second type of intermolecular interactions within the crystal, Hirshfeld surfaces and two-dimensional fingerprint plots were generated using *Crystal Explorer 17* software.^22^ The analysis of intermolecular contacts based on *d*_norm_ values reveals predominant parallel offset interactions^23,24^ between hydrogen and carbon atoms of the anthracenyl fragments (highlighted in red in Figure 3a), suggesting the presence of π–π stacking interactions. From the fingerprint plot analysis (Figure 3b–d and Table S1), it is evident that, apart from H···H contacts, the most significant interactions are of the H···C type (21.6%), while C···C contacts account for only 4.7%.

**Figure 3:**
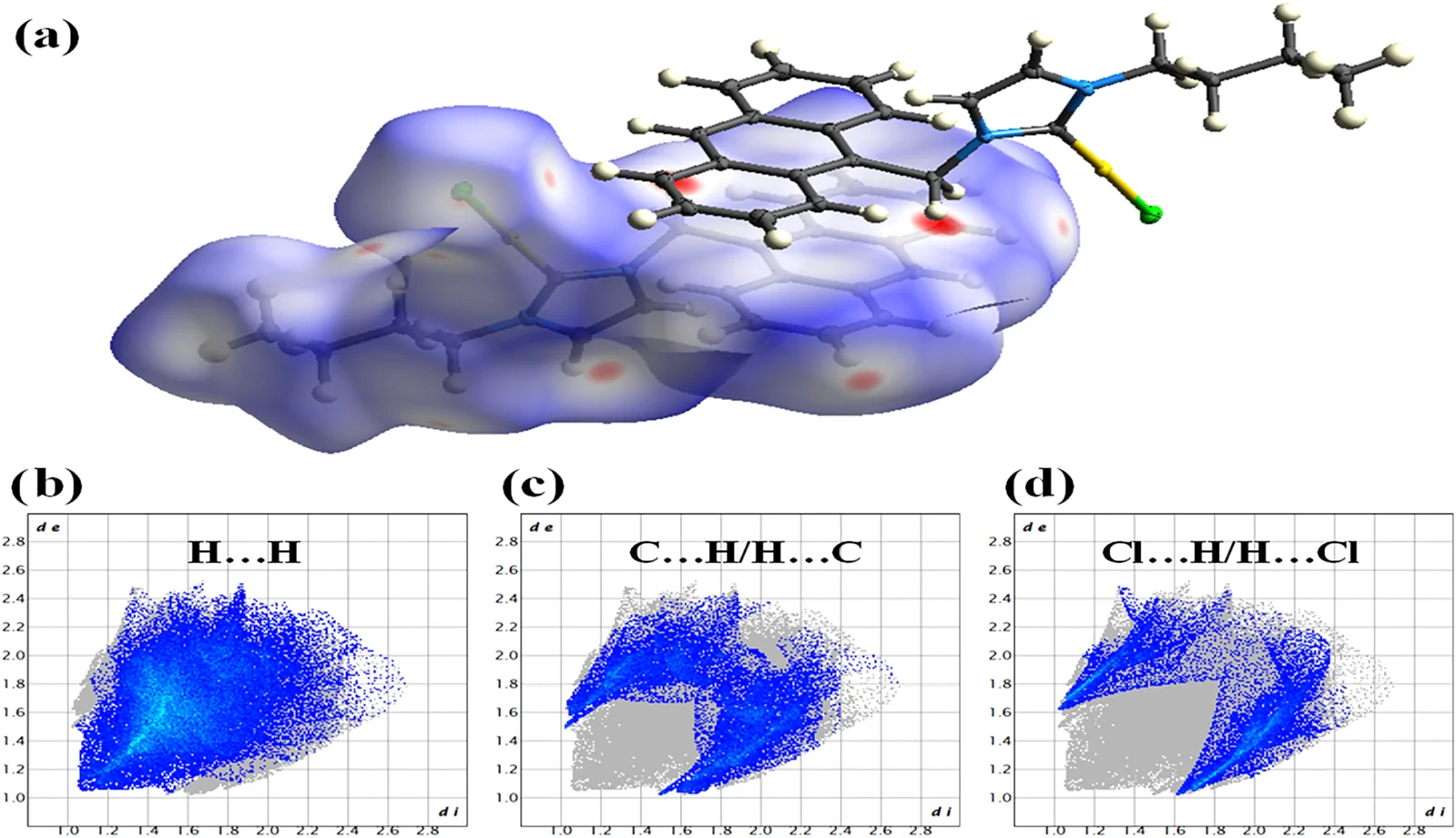
(a) Views of the Hirshfeld surface mapped over *d*_norm_ for anthracenyl (internal)-anthracenyl (external) contacts. The two-dimensional fingerprint plots for (b) H···H (51.8%), (c) C···H/H···C (21.6%), (d) Cl···H/H···Cl (14.3%) contacts.

Further confirmation comes from the noncovalent interaction (NCI) plot generated with *Multiwfn 3.8*, which reveals weak intermolecular interactions between the extended aromatic systems of adjacent molecular fragments (Figure S11). The green isosurfaces represent regions dominated by weak van der Waals interactions, confirming the presence of dispersive π···π stacking interactions between the aromatic moieties. The absence of intense blue regions further supports the conclusion that dispersion forces are the only contributors to the observed supramolecular packing motif.

Compound **4** had already been reported in the literature, where it was synthesized through transmetalation from the corresponding silver biscarbene.^25^ However, in this work compound **4** was prepared by deprotonation of the ligand **13**, carried out using LiHMDS in anhydrous *N*,*N*-dimethylformamide (DMF), and subsequent addition of 0.5 equiv of [AuCl­(SMe_2_)] drop by drop. After 4 h, the mixture was filtered to remove the formed lithium salts. The yellow solution was concentrated and diethyl ether was added, leading to the precipitation of the product as a yellow solid (yield: 64%). The same synthetic route was attempted starting from **14** for the preparation of a biscarbene bearing the BODIPY label, but unfortunately in this case it was not possible to obtain the desired compound.

The main advantage of this procedure over the previously reported silver-mediated route is that it provides direct access to the cationic bis-carbene Au­(I) compound from the imidazolium precursor, without requiring preparation and isolation of the corresponding silver biscarbene intermediate. This reduces the number of synthetic steps and minimizes the risk of residual silver contamination, an important aspect for compounds intended for biological studies. Moreover, the use of ligand **13** as a common precursor for both the monocarbene complex **3** and the bis-carbene compound **4** enables a direct comparison between neutral and cationic Au­(I)–NHC species bearing the same anthracenyl fluorescent tag.

### 2.2 Water Solubility and Log *P* Determination

The hydrophilic/lipophilic nature of compounds is crucial for their use in biology. Indeed, the solubility of a drug and its log *P* value can affect its mode of administration, absorption and distribution, as well as its general pharmacokinetic properties.^26^ The water solubility of the synthesized complexes was assessed by dissolving each compound in a 6.94 mM solution of dimethyl sulfone (DMSO_2_) in D_2_O, using DMSO_2_ as an internal standard for ^1^H NMR analysis. However, only signals corresponding to DMSO_2_ (3.13 ppm) and residual water (4.94 ppm) were observed (Figure S12), indicating negligible solubility of the complexes. The strong hydrophobic character of the aromatic fluorescent probes likely accounts for this poor solubility, making it impossible to evaluate their water solubility through ^1^H NMR determination. The lipophilic character of the complexes was further studied with the evaluation of their partition coefficient between water and n-octanol. The quantity of each complex in both water and octanol was evaluated through UV–vis absorption spectroscopy and the partition coefficient was expressed as Log *P* = log­(Absorption_octanol_/Absorption_water_). For complexes **4** and **5** (Figure 4b,c) Log *P* was calculated using the absorption peaks of the fluorescent labels at 369 and 527 nm, respectively. However, complex **3** exhibits a much lower absorption intensity (Figure 4a), hindering the detection of the anthracene signal in the aqueous phase. Therefore, the absorption peak at 256 nm, attributable to a metal-to-ligand charge-transfer (MLCT) transition from the gold center to the *p*_*z*_ orbital of the carbene carbon was used for the LogP determination.^27–29^

**Figure 4:**
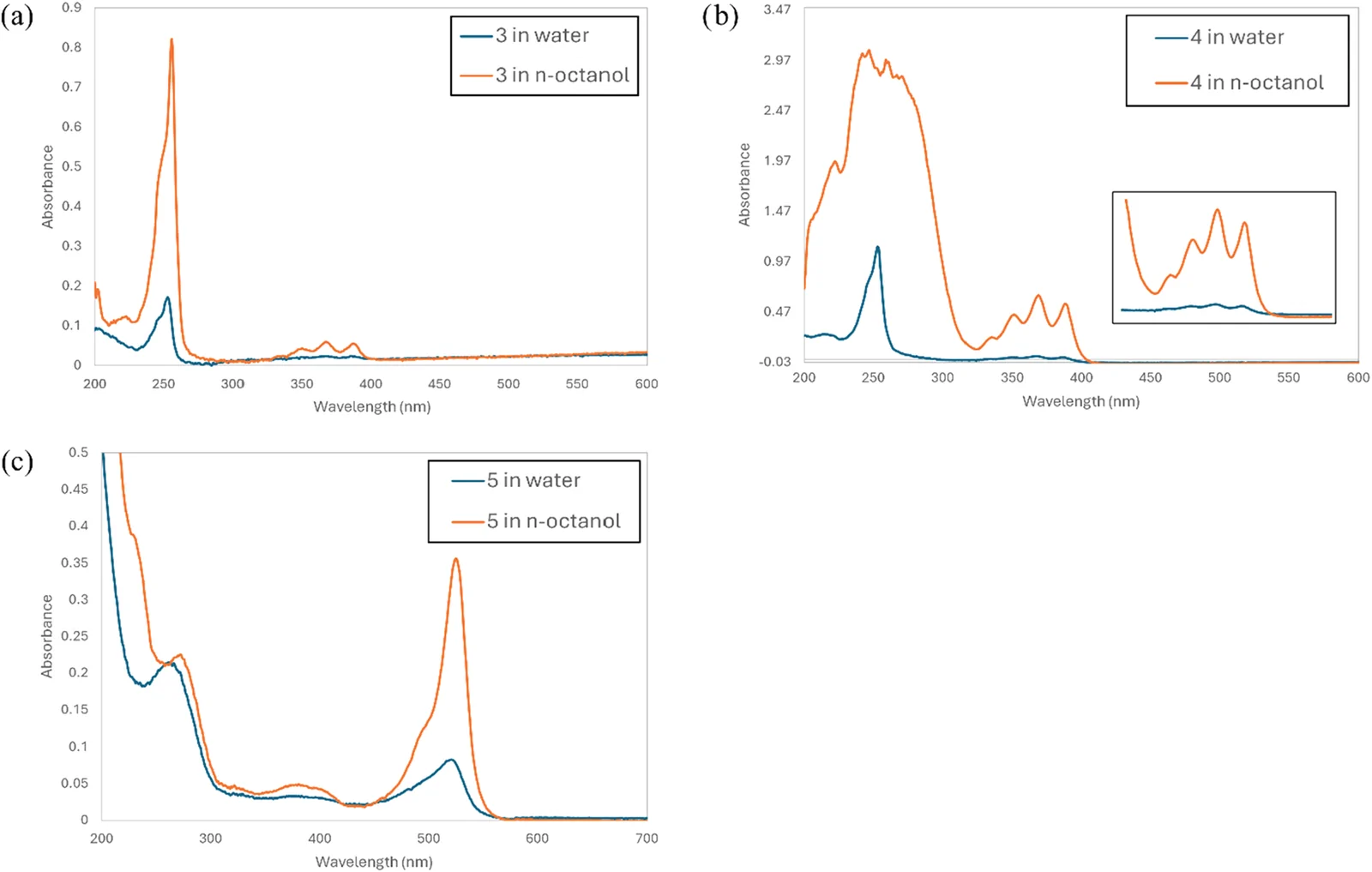
UV–vis absorption spectra of **3** (a), **4** (b), and **5** (c) in water (blue) and *n*-octanol (orange).

Although all complexes exhibit a pronounced lipophilic character, with all three having a Log *P* > 2, they still comply with Lipinski’s rule of five.^30^ Comparing the obtained LogP values with those of reference complexes **1** and **2** reported in the literature,^31^ an increase in lipophilicity is observed, especially concerning the charged complexes (Table 1). The substitution of the methyl group with a bulky aromatic moiety, such as anthracene, markedly decreases the overall hydrophilic character associated with the cationic framework. As a consequence, compound **4** displays the highest lipophilicity within the synthesized series, despite bearing a positive charge.

**Table 1:** Log *P* Values for the Reference and Synthesized Complexes

Complex	Log *P*
**1**	2.12 ± 0.11
**2**	0.06 ± 0.02
**3**	2.7 ± 0.2
**4**	2.9 ± 0.2
**5**	2.9 ± 0.3

### 2.3 Stability in Solution and Interaction Studies with Human Serum Albumin

#### 2.3.1 In Solution Stability

Stability in a biological environment is a fundamental requirement for compounds intended for biological use. In particular, antitumor complexes must be sufficiently stable to reach the target cells without undergoing deactivation or exerting toxic effects.^32^ Degradation within the bloodstream can also lead to the formation of biologically active or toxic compounds.^33^ The stability of the prepared complexes in both DMSO and a phosphate-buffered saline (PBS) solution was tested by means of UV–vis absorption spectroscopy (Figures S13–S16). UV–vis absorption spectra of the complexes in DMSO (Figure 5a) prove their stability for at least 48 h at 25 °C. Given their scarce solubility in water, the PBS solutions were prepared by adding 5 μL of a 10^–2^ M solution of each complex in DMSO to reach 1 mL in volume, obtaining 5 × 10^–5^ M solutions. The UV–vis absorption spectra, registered every 30 min for 48 h at 25 °C, show a rapid decrease in the intensity of the signals from the various complexes (Figure 5b). However, the formation of new bands was not observed, suggesting that the decrease in signal intensity is mainly due to precipitation of the compounds rather than their degradation.

**Figure 5:**
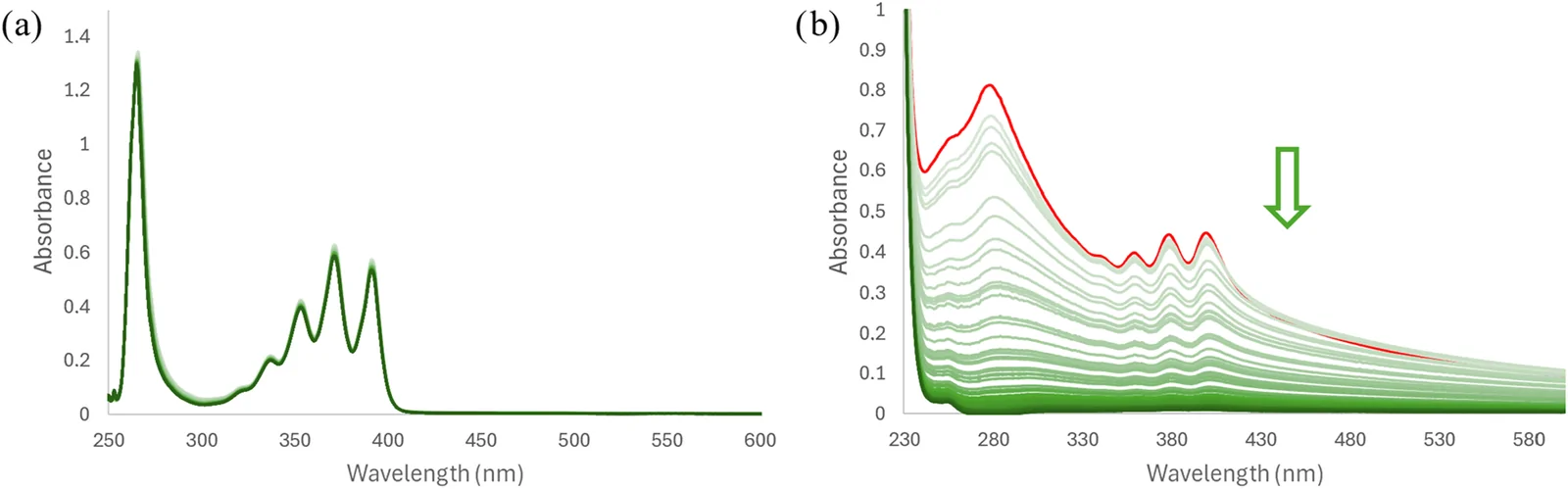
UV–vis absorption spectra of **3** in DMSO (a) and PBS (b) at 25 °C at *t* = 0 h (red) and over 48 h (green).

#### 2.3.2 UV–Vis Interaction Studies with Human Serum Albumin

Human serum albumin (HSA) is the most abundant protein in the bloodstream. Therefore, understanding the interaction between gold­(I) compounds and HSA is crucial for grasping the pharmacokinetics of these metal-based complexes. Gold­(I) antiarthritic drugs, especially auranofin, are well-known for their ability to bind to cysteine-34 of HSA and use the protein as a carrier.^34^ For this reason, UV–vis absorption spectra of the synthesized complexes in the presence of HSA in PBS were registered at 37 °C over a 24 to 48 h time period. The experiments were conducted with a 1:1 protein-to-metal ratio, monitoring changes in the complexes’ absorption spectra (Figure S17). While the anthracene-bearing carbenes did not show the formation of new signals (Figure 6a), the disappearance of the scattering contribution, coupled with the compounds’ increased stability over time compared to the spectra of the single complexes previously acquired for stability experiments in PBS (Figure 5b), suggests that HSA stabilizes the compounds in an aqueous solution. Complex **5**, instead, exhibited an increasing signal at 526 nm and an isosbestic point at 536 nm, as well as a decrease in the signal at 286 nm (Figure 6b). The formation of this new signal and the presence of the isosbestic point in both spectra indicate the formation of a new species resulting from binding between the complex and the protein.

**Figure 6:**
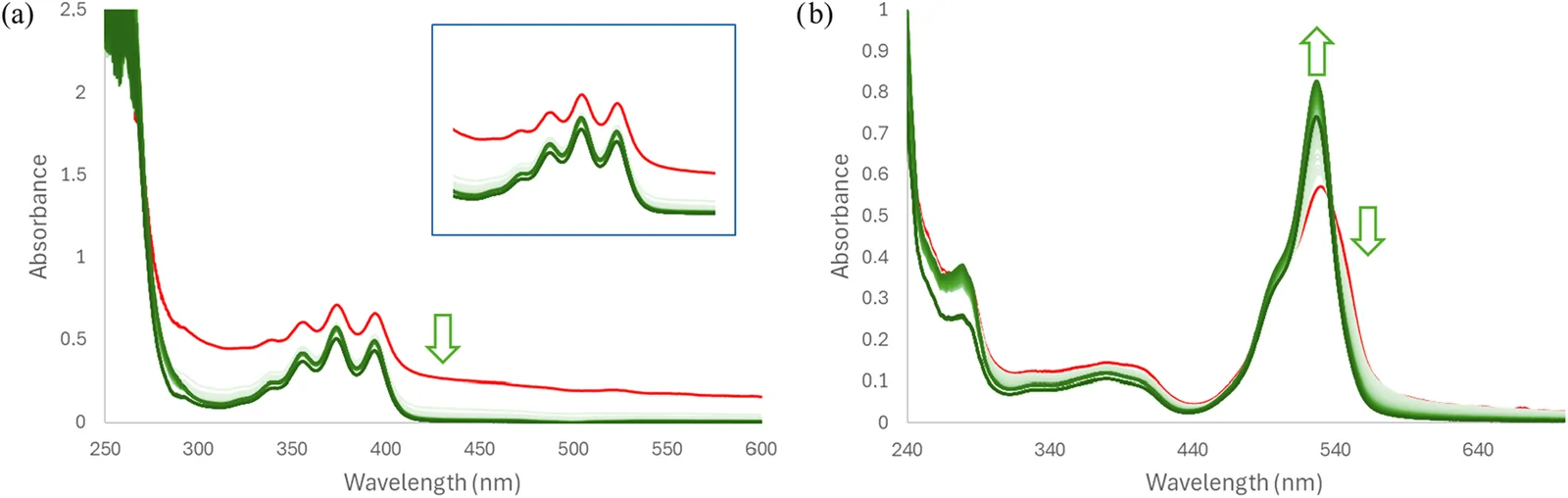
UV–vis absorption spectra of **4** (a) and **5** (b) incubated with HSA (1:1 metal to protein) in PBS at 37 °C at *t* = 0 h (red) and over 48 h (green).

#### 2.3.3 ESI-MS Interaction Studies with HSA

ESI-MS experiments were then performed to assess whether the spectroscopic changes observed in the presence of HSA corresponded to the formation of covalent metallodrug–protein adducts. As shown in Figure 7, the ESI-MS spectra of the conjugates were compared with those of a native HSA control. The control spectrum of free human serum albumin displays three primary peaks consistent with our previous findings^35,36^ (Figure 7a). The signal at 66 438 Da matches the theoretical mass of the HSA sequence. The two higher-mass peaks correspond to the post-translational modifications (PTMs) inherent to the commercial protein stock: the peak at 66 556 Da is attributed to cysteinylated HSA, while the peak at 66 718 Da represents glycosylated species. A minor, unassigned signal was also observed at 66 471 Da.

**Figure 7:**
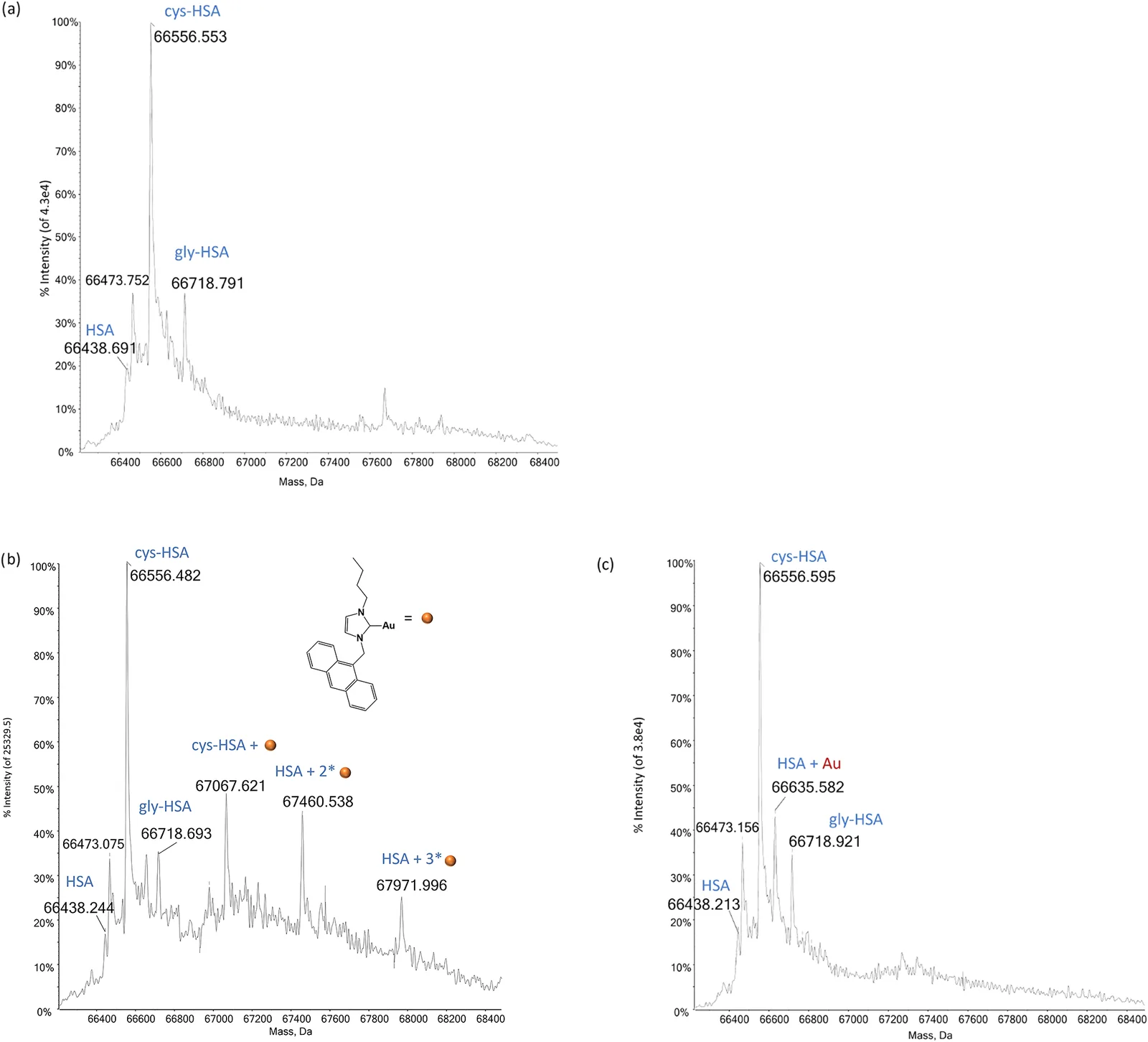
Deconvoluted ESI-Q-TOF spectra of (a) nonmetalated HSA and the adducts with (b) complex **3** and (c) complex **5** in a 1:1 protein to gold complex ratio at 24 h.

Notably, of the three metal-based drugs tested, only complexes **3** and **5** produced significant alterations to the ESI-MS spectrum of the native protein. In contrast, no adduct formation was observed when free HSA reacted with complex **4**, likely due to the inherent stability of the gold-carbene bond (Figure S18).

Following incubation with complex **3** (Figure 7b), HSA exhibited three new signals: the first is at 67 067 Da (+511 Da), which belongs to cysteinylated HSA conjugated with a single 1-(anthracen-9-ylmethyl)-3-butylimidazol-2-ylidene gold­(I) moiety; a second peak appears at 67 460 Da (+1022), consistent with the interaction of two aforementioned gold-carbene fragments; and a lower-intensity signal, at 67 971 (+1533) Da indicating the further addiction of a third gold-carbene fragment.

In contrast, the spectrum obtained from the reaction with complex **5** exhibited significant differences (Figure 7c); while the original peaks from the control HSA spectrum remained detectable, a new signal emerged at 66 635 Da (+197 Da). This shift corresponds to the addition of a bare gold­(I) ion to the HSA sequence.

These findings are in agreement with the UV–vis spectroscopic data regarding the interaction between the gold compounds and HSA. Specifically, the interaction between the protein and the 1-(anthracen-9-ylmethyl)-3-butylimidazol-2-ylidene gold moiety could explain the enhanced aqueous solubility of complexes **3** and **4**. More precisely, for compound **3**, mass spectrometry highlights a covalent interaction between HSA and the 1-(anthracen-9-ylmethyl)-3-butylimidazol-2-ylidene gold moiety, with loss of the chloride anion. On the other hand, compound **4**, as can be deduced from Figure S18, interacts with the protein only through weak noncovalent interactions. Conversely, the pronounced spectral variations observed for the HSA-complex **5** adduct -characterized by the appearance of a new absorption band and a well-defined isosbestic point- can be ascribed to the total displacement of the original ligands from the metal center, facilitating the sequestration of a bare gold­(I) ion as identified by mass spectrometry.

### 2.4 Biological Studies

#### 2.4.1 Cell Viability Studies

The cytotoxicity of the compounds was evaluated via the MTT assay in the human ovarian cancer cell line A2780, either sensitive (A2780/S) or resistant to cisplatin (A2780/R), after 72 h of incubation. The poor solubility of **5** has prevented its use at concentrations orders of magnitude higher than μM, resulting in unreliable values. The obtained IC_50_ values are reported in Table 2, with those relative to the reference complexes **1** and **2** reported in the literature.^37^ Auranofin and cisplatin are also reported as references.

**Table 2:** Measured IC_50_ Values (μM) Obtained from Cytotoxicity MTT Assay after a 72 h Incubation Period

Complex	A2780/S IC_50_ (μM) ± SD^a^	A2780/R IC_50_ (μM) ± SD^a^
**Cisplatin**	1.5 ± 0.1	16.7 ± 0.6
**Auranofin**	0.8 ± 0.2	3.9 ± 0.3
**1**	3.5 ± 0.9	5.2 ± 0.5
**2**	0.10 ± 0.01	0.75 ± 0.05
**3**	7.8 ± 0.8	3.2 ± 0.4
**4**	1.8 ± 0.4	0.7 ± 0.2
**5**	nd	nd

a Values are mean ± standard deviation (SD) of three biological independent experiments.

As stated above, it was not possible to determine the IC_50_ value of complex **5** due to its poor solubility. It was, in fact, not feasible to obtain solutions at higher concentrations needed for the dose-inhibition curve, due to the precipitation of the compound. This led to the recording of only one death point, making it impossible to accurately determine the IC_50_ of the complex. Although the introduction of the anthracenyl residue leads to higher IC_50_ values compared with the parent complexes in A2780/S cells, the same structure–activity trend is retained: the cationic bis-carbene compound **4** is markedly more active than the corresponding neutral monocarbene complex **3**. This behavior is consistent with previous reports on Au­(I)–NHC systems, in which cationic and highly lipophilic bis-carbene or related gold carbene scaffolds generally show enhanced cellular bioavailability and stronger antiproliferative effects.^38^ In particular, lipophilic cationic Au­(I)–NHC complexes have been rationally designed as mitochondria-targeted agents, because their charge and hydrophobicity favor accumulation driven by the mitochondrial membrane potential. This concept is well established for mitochondria-targeted Au­(I)–NHC and related gold carbene complexes, including dinuclear and bis-carbene derivatives, for which mitochondrial effects, cellular uptake, and antiproliferative activity were found to be closely interconnected. In the present series, the higher activity of compound **4** is therefore in line with its cationic character, bis-carbene structure, and higher Log *P* value compared with compound **3**.^39^

Moreover, complexes **3** and **4** have been shown to be more active on cisplatin-resistant cell lines than on sensitive ones. Specifically, complex **3** exhibits cytotoxic activity comparable to auranofin, whereas complex **4** shows higher activity. Rather, the data indicate that compounds **3** and **4** retain significant antiproliferative activity after introduction of the fluorescent tag, while showing a slightly different potency compared with the corresponding parent complexes.

It should also be considered that the cytotoxicity assays were performed in complete RPMI-1640 medium supplemented with 10% fetal bovine serum, and therefore not under protein-free conditions. Thus, during cell treatment, the compounds were exposed to serum proteins that may influence their apparent solubility, speciation, and cellular availability. This aspect is particularly relevant for poorly water-soluble Au­(I)–NHC derivatives and for compounds able to interact with albumin. However, serum protein binding should not necessarily be interpreted only as immobilization, since albumin may also contribute to transport and stabilization of gold species in biological media, as previously reported for auranofin and related Au­(I) compounds.^40,41^ Therefore, the present cytotoxicity data should be regarded as evidence of antiproliferative activity under serum-containing *in vitro* culture conditions, rather than as a direct predictor of *in vivo* pharmacokinetic behavior. In this context, cisplatin was used as a clinically established metallodrug reference in A2780/S and A2780/R cells, although its speciation, protein-binding profile, and mechanism of action are clearly distinct from those of Au­(I)–NHC compounds.

Moreover, it should be noted that these cytotoxicity data do not allow us to rule out a possible contribution of the anthracenyl fragment to biomolecular recognition, including DNA interactions. Anthracene derivatives and other planar polycyclic aromatic systems may, in principle, interact with DNA through intercalative or related binding modes.^42,43^ Therefore, these data should not be interpreted as evidence that the anthracenyl probe does not alter the biological or pharmacokinetic profile of the parent Au­(I)–NHC scaffolds.

#### 2.4.2 Uptake Studies through Fluorescence-Activated Cell Sorting Flow Cytometry

The fluorescent properties of the synthesized compounds were exploited to assess their cellular uptake by fluorescence-activated cell sorting (FACS) flow cytometry. In particular, uptake of compounds **3** and **4** was evaluated in cisplatin-sensitive A2780/S cells and in cisplatin-resistant A2780/R cells. The fluorescence signal detected for these compounds can be reasonably assigned mainly to the anthracenyl fluorophore. However, coordination to the Au­(I)–NHC fragment may perturb the emissive response through changes in the electronic structure, conformational rigidity, local environment, and intermolecular interactions. In related Au­(I)–NHC systems, metal coordination has been reported to affect emission intensity and spectral profile, sometimes through metal-perturbed ligand-centered states or charge-transfer contributions.^44,45^ In addition, the heavy-atom effect associated with Au­(I) may promote intersystem crossing, leading to partial fluorescence quenching or to longer-lived emissive states, depending on the ligand environment and aggregation state.^46^

Under the experimental conditions used here, compounds **3** and **4** showed a detectable emission in the Pacific Blue channel (421 nm), indicating that incorporation of the Au­(I)–NHC fragment does not suppress the fluorescence required for cellular tracking (Figure S19). By contrast, compound **5** displayed a broad fluorescence response across several detection channels, with no selective emission in the Pacific Blue channel, making it less suitable for FACS-based biodistribution studies.

Both anthracene-labeled compounds were efficiently internalized by the cells, with compound **4** producing a markedly higher fluorescence signal than compound **3** (Figure 8). This behavior is consistent with the presence of two anthracenyl units in the bis-carbene compound and with its higher cellular uptake, likely favored by its cationic character and higher lipophilicity. Compound **4** has also shown a far greater internalization in A2780/R cells with respect to the cisplatin-sensitive ones. While less pronounced, the uptake of complex **3** was higher in A2780/R. This confirms that both complexes exhibited stronger cytotoxicity, with the most significant impact on the platinum-resistant cell line.

**Figure 8:**
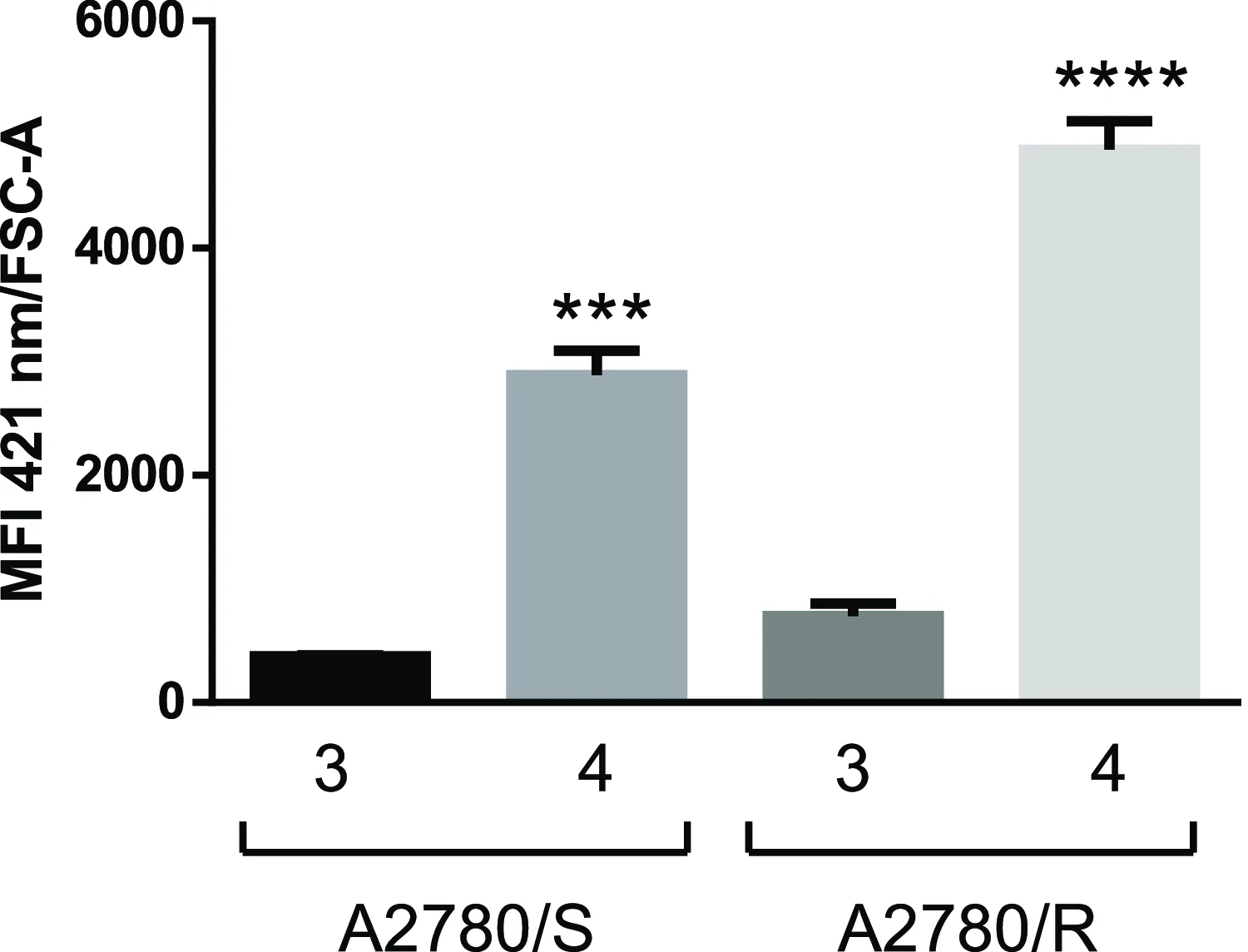
Cellular uptake of **3** and **4** in A2780/S and A2780/R cancer cells using cytofluorimetric analysis. The histogram reports the mean and the standard deviation of three independent experiments.

However, the fluorescence intensities measured in cells should not be interpreted as a direct comparison of intrinsic photophysical efficiency, since they also depend on cellular uptake, intracellular concentration, and microenvironmental effects.

#### 2.4.3 Biodistribution Studies through Cell Fractionation Method

To quantitatively evaluate the subcellular localization of complex **3** and **4** compounds, we employed a cellular fractionation approach leveraging their intrinsic fluorescent properties due to the anthracenyl residue. Following incubation with the compounds, A2780/S cells were partitioned into cytosolic and mitochondrial fractions. The fluorescence intensity of the compounds within these isolated fractions was then measured using a microplate reader. The results are summarized in Figure 9, panel A.

**Figure 9:**
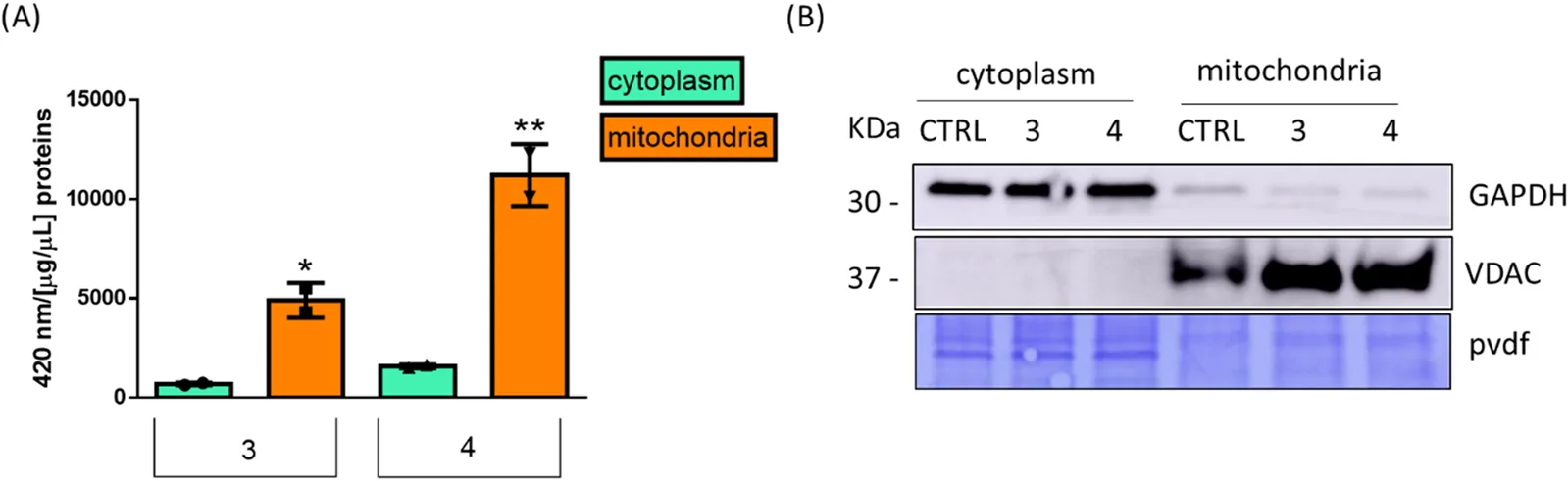
(A) Cellular distribution of complexes **3** and **4** within the cytosol and mitochondria. A2780/S treated with complex **3** and **4** were fractionated into cytoplasm and mitochondria. The fluorescent intensity of these fractions was evaluated using a multireader plate. The histogram reports the mean and the standard deviation of three independent experiments; (B) Western blot analysis on cytosolic and mitochondrial fractions: GAPDH and VDAC, markers of the cytosolic and mitochondrial fractions, respectively, were used to validate the fractionation method. The pvdf membrane was stained with blue Coomassie to ensure the loading of the proteins in each fraction.

On the whole, consistent with FACS data, we observed a greater cellular uptake of complex **4**.

Notably, both **3** and **4** preferentially accumulated within the mitochondrial fraction, with compound **4** showing the highest accumulation. This behavior is consistent with previous studies on mitochondria-targeted lipophilic cationic Au­(I)–NHC complexes.^47,48^ However, since subcellular distribution was assessed through the fluorescence signal of the anthracenyl-labeled compounds, a possible contribution of the fluorophore to the observed localization cannot be excluded. Thus, these data should be interpreted as preferential mitochondrial accumulation of the fluorescent Au­(I)–NHC species under the applied experimental conditions, while further experiments would be required to confirm that mitochondrial targeting is independent of the fluorescent tag.^49^

Furthermore, Western blot analysis was performed on the cytosolic and mitochondrial fractions to ensure the absence of significant cross-contamination between organelles (Figure 9, panel b).

## 3 Conclusions

An interesting strategy to gain valuable mechanistic information on novel anticancer metallodrugs and track their behavior and fate within biological systems is to insert a fluorescent probe into their scaffold. The development of multifunctional metallodrugs that can act as molecular probes is an emerging and powerful approach in medicinal inorganic chemistry. A few research groups worldwide have previously pursued this strategy with remarkable results.^47,48^

This study involved designing and synthesizing a small panel of gold­(I) *N*-heterocyclic carbene complexes that either bear anthracenyl or BODIPY fluorophores. All of the compounds in the panel were characterized using ^1^H and ^13^C nuclear magnetic resonance (NMR) spectroscopy and elemental analysis. The ^19^F NMR spectrum of compound **5** was recorded, and compound **3** was successfully crystallized and structurally characterized by X-ray diffraction. UV–vis spectroscopy was employed to conduct lipophilicity studies, revealing high LogP values for all the compounds, especially the cationic complex **4**. Compared to the reference gold carbenes **1** and **2** (Figure 1), the synthesized mono- and bis-carbenes exhibited opposite trends in lipophilic behavior.

Following extensive characterization, the three complexes were evaluated for their behavior and reactivity in solution. The complexes remained stable in dimethyl sulfoxide (DMSO) at 25 °C for 48 h. However, under the same conditions in phosphate-buffered saline (PBS), they gradually precipitated. Interaction studies with HSA revealed compound-dependent behavior. For compound **3**, ESI-MS data support the formation of covalent HSA adducts retaining the Au-NHC fragment, whereas compound **4** appears to be mainly stabilized in solution through weak noncovalent interactions, without detectable covalent adduct formation under the applied ESI-MS conditions. Conversely, compound **5** undergoes a different process involving ligand displacement and formation of protein-bound gold species. Thus, HSA does not simply “protect” all compounds from degradation, but rather modulates their speciation, solubility, and protein-binding behavior in a compound-specific manner. Then, some of their biological and pharmacological properties were assessed. Overall, the cell viability analysis showed that the anthracenyl-labeled complexes retained relevant cytotoxic activity, although their potency differed from that of the corresponding parent Au­(I)–NHC compounds. In line with the reference panel, compound **4** exhibited higher cytotoxicity than compound **3**. In A2780/S cells, a clear reduction in antiproliferative activity was observed when comparing compound **3** to **1**, as well as compound **4** to **2**, yet their IC_50_ values remained within the low micromolar range. Interestingly, both compounds **3** and **4** were found to be more active against the cisplatin-resistant cell line than the sensitive one. Compound **4** was even more cytotoxic than auranofin. The IC_50_ value of compound **5** could not be precisely calculated due to its low solubility. Nevertheless, it exhibited lower cytotoxic activity than the other two complexes, suggesting that substitution with the anthracenyl moiety influences the neutral compound more than the cationic one.

Cellular uptake studies in A2780 ovarian cancer cells confirmed the feasibility of monitoring the progressive internalization of both fluorescent compounds via FACS analysis. Overall, consistent with its higher cytotoxicity, compound **4** exhibited greater accumulation than **3**. Interestingly, the uptake of both compounds was further enhanced in cisplatin-resistant A2780 cells, probably leading to the enhanced cytotoxicity of both compounds observed in this cell line. Notably, the ability to directly correlate intracellular accumulation, subcellular distribution, and biological activity highlights the added value of fluorescent tagging in mechanistic studies of metallodrugs, provided that the possible influence of the fluorophore itself on cellular behavior is carefully considered.

Furthermore, biodistribution assays showed preferential accumulation of both complexes in the mitochondria, validating these organelles as the primary site of action for gold carbenes.^50^

In conclusion, we have demonstrated that incorporating a fluorescent probe into the metallodrug scaffold is an effective approach for tracking metallodrugs in cells, but also a versatile strategy to enable metallomics and metalloproteomics investigations aimed at identifying molecular targets and perturbed cellular pathways. Of the two probes considered, we demonstrated that the BODIPY probe had several disadvantages, including more difficult synthesis and purification, an inability to synthesize the corresponding bis-carbene, a broad fluorescent spectrum, and low cytotoxic activity. By contrast, anthracenyl complexes exhibited easier synthesis and substantially unaltered cytotoxic effects with respect to the parent compounds. Thus, anthracene emerged as the more suitable probe for the present proof-of-concept study, although it should not be regarded as a biologically inert tag. Rather, possible effects of the anthracenyl fragment on lipophilicity, biomolecular recognition, and intracellular distribution must be considered when interpreting the biological data. Overall, this study provides a proof-of-concept for the rational design of fluorescently labeled gold complexes as chemical tools to bridge the gap between metal-based drug development and systems-level mechanistic investigations. Importantly, while our biological evaluations establish a solid baseline profile and showcase the ability of these derivatives to overcome cisplatin resistance, they represent a preliminary in vitro screening. Further extensive investigations, specifically focused on assessing the selectivity index of these gold­(I) complexes against nontumorigenic cell lines to define their exact therapeutic window, are currently planned as the essential next phase of this research.

## 4 Experimental Section

### 4.1 Chemistry

#### 4.1.1 Materials and Methods

The reactions, unless otherwise specified, were performed without air exclusion. All the reagents were purchased from Sigma-Aldrich and used without further purification, except for triethylamine, which was distilled under reduced pressure and dried over molecular sieves. [AuCl­(SMe_2_)] was synthesized following a procedure reported in the literature.^51^ NMR spectra were collected at room temperature on NMR JEOL YZ400 MHz and NMR JEOL CZR 500 MHz spectrometers. The elemental analysis was carried out with a Vario Micro Cube CHNOS Elemental Analyzer by Elementar. UV–Vis spectra were recorded on an Agilent Cary 60 spectrophotometer. Phosphate-buffered saline (sodium phosphate = 20 mM, sodium chloride = 100 mM, pH = 7.4) used for the stability studies was prepared by dissolving a suitable amount of the respective solid salt in ultrapure water. Lyophilized HSA was purchased from Merck and used without further purification or manipulation. A stock solution (10^–3^ M) of HSA was prepared by dissolving a known quantity of the solid in ultrapure water. RPMI 1640 cell culture medium, fetal bovine serum (FBS), and phosphate-buffered saline (PBS) used for cytotoxic assays were obtained from Euroclone (Milan, Italy).

#### 4.1.2 Synthesis

##### 4.1.2.1 Synthesis of Compound **10** (4,4-Difluoro-8-(4-chloromethylphenyl)-1,3,5,7- tetramethyl-2,6-diethyl-4-bora-3a,4a-diaza-*s*-indacene)

4-(chloromethyl)­benzoyl chloride (**7**) (204 mg, 1.08 × 10^–3^ mol) and 3-ethyl-2,4-dimethylpyrrole (**8**) (291 μL, 2.16 × 10^–3^ mol) were dissolved in anhydrous CH_2_Cl_2_ under inert atmosphere. The red solution was stirred and refluxed for 6 h and kept under stirring at room temperature overnight. NEt_3_ (1.5 mL, 1.1 × 10^–2^ mol) was added to the solution and stirred for 15 min before adding BF_3_ × Et_2_ (1.5 mL, 1.2 × 10^–2^ mol). The mixture was stirred for 1 h and then washed with water and HCl (10%). The organic phases were collected and dried over anhydrous Na_2_SO_4_. The crude product was purified using silica gel column chromatography (eluent CH_2_Cl_2_/hexane 1:1). The yellow fractions were collected, and the solvent was removed, obtaining a red solid (74 mg, yield: 16%). ^1^H NMR (400 MHz, CDCl_3_) δ (ppm): 7.50 (d, *J* = 8.0 Hz, 2H, Benz), 7.28 (d, *J* = 8.0 Hz, 2H Benz), 4.66 (s, 2H, BenzC**H**_**2**_Cl), 2.52 (s, 6H, PyC**H**_**3**_), 2.29 (q, *J* = 7.5 Hz, 4H, PyC**H**_**2**_CH_3_), 1.31 (s, 6H, Py**CH**_**3**_), 0.97 (t, *J* = 7.5 Hz, 6H, PyCH_2_C**H**_**3**_).

##### 4.1.2.2 Synthesis of Compound **13** (1-(Anthracen-9-ylmethyl)-3-butylimidazolium Chloride)

9-(chloromethyl)­anthracene­(**12**) 98% (462 mg, 1.99 × 10^–3^ mol) and 1-butylimidazole (**11**) (270 μL, 2.05 × 10^–3^ mol) were dissolved in 40 mL of acetonitrile, obtaining a yellow solution. The mixture was refluxed at 80 °C with an air cooler for 48 h. The solvent was removed under reduced pressure, obtaining a yellow-pale solid (595 mg, yield: 85%). ^1^H NMR (400 MHz, CDCl_3_) δ (ppm): 11.19 (s, 1H, NCHN), 8.52 (s, 1H, Ant H8), 8.33 (d, *J* = 8.7 Hz, 2H, Ant H6–H10), 8.01 (d, *J* = 8.7 Hz, 2H, Ant H3–H13), 7.61 (m, 2H, Ant H5–H11), 7.48 (m, 2H, H4–H12), 7.06 (m, 1H, Im), 6.65 (m, 1H, Im), 6.63 (s, 2H, AntC**H**_**2**_Im), 4.25 (t, *J* = 7.5 Hz, 2H, NC**H**_**2**_CH_2_CH_2_CH_3_), 1.82 (m, 2H, NCH_2_C**H**_**2**_CH_2_CH_3_), 1.32 (m, 2H, NCH_2_CH_2_C**H**_**2**_CH_3_), 0.89 (t, *J* = 7.4 Hz, 3H, NCH_2_CH_2_CH_2_C**H**_**3**_). ^13^CNMR (400 MHz, CDCl_3_) δ (ppm): 137.74 (N**C**N), 131.33 (Ant **C7**), 130.94 (Ant **C2**), 130.61 (Ant **C8**), 129.59 (Ant **C3/C6**), 128.35 (Ant **C6/C3**), 125.63 (Ant **C4/C5**), 122.84 (Ant **C5/C4**), 122.10­(Im), 121.63 (Ant **C1**), 121.07 (Im), 50.08 (Ant**C**H_2_Im), 45.61 (N**C**H_2_CH_2_CH_2_CH_3_), 32.09 (NCH_2_ **C**H_2_CH_2_CH_3_), 19.54 (NCH_2_CH_2_ **C**H_2_CH_3_), 13.44 (NCH_2_CH_2_CH_2_ **C**H_3_)

##### 4.1.2.3 Synthesis of Compound **14** (1-(4,4-Difluoro-8-(4-methylphenyl)-1,3,5,7-tetramethyl-2,6-diethyl-4-bora-3a,4a-diaza-s-indacene)-3-butylimidazolium Chloride)

**10** (93 mg, 2.2 × 10^–4^ mol) was dissolved in 4.0 mL (3.0 × 10^–2^ mol) of **11**. The yellow solution was stirred for 24 h and then concentrated. Diethyl ether was added to obtain a red solid (84 mg, yield: 68%). ^1^H NMR (400 MHz, CDCl_3_) δ (ppm): 11.23 (s, 1H, NC**H**N), 7.62 (d, *J* = 8.0 Hz, 2H, Benz), 7.33 (d, *J* = 8.0 Hz, 2H, Benz),7.23 (s, 1H, Im), 7.17 (s, 1H, Im), 5.79 (s, 2H, BenzC**H**_**2**_Cl), 4.31 (t, *J* = 7.4 Hz, 2H, NC**H**_**2**_CH_2_CH_2_CH_3_), 2.50 (s, 6H, PyC**H**_**3**_), 2.26 (q, *J* = 7.5 Hz, 4H, PyC**H**_**2**_CH_3_), 1.92 (m, 2H, NCH_2_C**H**_**2**_CH_2_CH_3_), 1.38 (m, 4H, NCH_2_CH_2_C**H**_**2**_CH_3_), 1.19 (s, 6H, Py**CH**_**3**_), 0.96 (t, *J* = 7.2 Hz, 3H, NCH_2_CH_2_CH_2_C**H**_**3**_ **)**, 0.95 (t, *J* = 7.6 Hz, 6H, PyCH_2_C**H**_**3**_).

##### 4.1.2.4 Synthesis of **3** (1-(Anthracen-9-ylmethyl)-3-butylimidazol-2-ylidene Gold Chloride)

Ag_2_O (65 mg, 2.8 × 10^–4^ mol) was added to a solution of **13** (142 mg, 4.00 × 10^–4^ mol) in anhydrous CH_2_Cl_2_ under inert atmosphere. The mixture was kept stirring for 20 h in the dark, after which it was filtered to remove unreacted Ag_2_O. [AuCl­(SMe_2_)] (109 mg, 3.70 × 10^–4^ mol) was added to the solution and kept under stirring for 3 h. The mixture was filtered through Celite to eliminate reduced gold. The solution was concentrated with rotavapor and diethyl ether was added to obtain a yellow precipitate (159 mg, yield: 78%). ^1^H NMR (400 MHz, CDCl_3_) δ (ppm): 8.55 (s, 1H, Ant H8), 8.22 (m, 2H, Ant H6–H10), 8.05 (m, 2H, Ant H3–H13), 7.54 (m, 4H, Ant H5–H11–H4-H12), 6.67 (d, *J* = 2.0 Hz, 1H, Im), 6.27 (*d*, J= 2.0 Hz, 1H, Im), 6.23 (s, 2H, AntC**H**_**2**_Im), 4.18 (*t*, J= 7.3 Hz, 2H, NC**H**_**2**_CH_2_CH_2_CH_3_), 1.81 (m, 2H, NCH_2_C**H**_**2**_CH_2_CH_3_), 1.35 (dq, *J* = 14.8 Hz, 7.4 Hz, 2H, NCH_2_CH_2_C**H**_**2**_CH_3_), 0.94 (t, *J* = 7.4 Hz, 3H, NCH_2_CH_2_CH_2_C**H**_**3**_). ^13^CNMR (400 MHz, CDCl_3_) δ (ppm): 170.48 (N**C**N), 131.44 (Ant **C7**), 131.00 (Ant **C2**), 130.05 (Ant **C8**), 129.61 (Ant **C3/C6**), 127.84 (Ant **C6/C3**), 125.55 (Ant **C5/C4**), 123.94 (Ant **C1**), 123.18 (Ant **C4/C5**), 120.31 (Im), 119.77 (Im), 51.54 (Ant**C**H_2_Im), 47.18 (N**C**H_2_CH_2_CH_2_CH_3_), 33.17 (NCH_2_ **C**H_2_CH_2_CH_3_), 19.77 (NCH_2_CH_2_ **C**H_2_CH_3_), 13.74 (NCH_2_CH_2_CH_2_ **C**H_3_). Elemental analysis for C_22_H_22_AuClN_2_ calculated: C 48.34%, H 4.06%, N 5.13%; experimental: C 48.26%, H 4.03%, N 5.09%.

##### 4.1.2.5 Synthesis of **5** (1-(4,4-Difluoro-2,6-diethyl-8-(4-methylphenyl)-1,3,5,7-tetramethyl-2,6-diethyl-4-bora-3a,4a-diaza-s-indacene)-3-butylimidazol-2-ylidene Gold Chloride)

**14** (45 mg, 8.0 × 10^–5^ mol) and Ag_2_O (9 mg, 4 × 10^–5^ mol) were dissolved in anhydrous CH_2_Cl_2_ under inert atmosphere. The mixture was kept stirring for 20 h in the dark, after which it was filtered to remove unreacted Ag_2_O. [AuCl­(SMe_2_)] (24 mg, 8.0 × 10^–5^ mol) was added to the solution, and it was kept under stirring for 4 h. The mixture was filtered through Celite, concentrated with rotavapor, and pentane was added to obtain a red precipitate (23 mg, yield: 65%). ^1^H NMR (400 MHz, CDCl_3_) δ (ppm): 7.38 (d, *J* = 8.2 Hz, 2H, Benz), 7.29 (d, *J* = 8.2 Hz, 2H, Benz), 7.17 (s, 1H, Im), 6.98 (s, 1H, Im), 5.79 (s, 2H, BenzC**H**_**2**_Cl), 4.31 (t, *J* = 7.4 Hz, 2H, NC**H**_**2**_CH_2_CH_2_CH_3_), 2.50 (s, 6H, PyC**H**_**3**_), 2.26 (q, *J* = 7.5 Hz, 4H, PyC**H**_**2**_CH_3_), 1.38 (m, 2H, NCH_2_C**H**_**2**_CH_2_CH_3_), 1.33 (m, 4H, NCH_2_CH_2_C**H**_**2**_CH_3_), 1.19 (s, 6H, Py**CH**_**3**_), 0.96 (t, *J* = 7.2 Hz, 3H, NCH_2_CH_2_CH_2_C**H**_**3**_), 0.95 (t, *J* = 7.6 Hz, 6H, PyCH_2_C**H**_**3**_). ^13^C NMR (400 MHz, CDCl_3_) δ (ppm): 171.49 (**C**-Au), 154.13 (BF_2_N**C**), 138.99 (BODIPY **C**), 138.20 (BODIPY **C**), 136.50 (BODIPY **C**), 136.10 (BODIPY **C**), 133.07 (Benz **C**), 130.67 (Benz **C**), 129.28 (Benz **C**H), 128.56 (Benz **C**H), 121.21 (Im), 120.22 (Im), 54.93 (N**C**H_2_Benz), 51.54 (BODIPY-**C**H_2_CH_3_), 33.14 (N**C**H_2_CH_2_CH_2_CH_3_), 19.77 (BODIPY-**C**H_3_), 17.15 (BODIPY-**C**H_3_), 14.69 (BODIPY-CH_2_ **C**H_3_), 13.73 (NCH_2_ **C**H_2_CH_2_CH_3_), 12.60 (NCH_2_CH_2_ **C**H_2_CH_3_), 11.91 (NCH_2_CH_2_CH_2_ **C**H_3_). ^19^F NMR (400 MHz, CDCl_3_) δ (ppm): 145.66 (dd, 2F, *J* = 66.6, 32.7 Hz). Elemental analysis for C_31_H_39_AuBClF_2_N_4_ calculated: C 49.71%, H 5.25%, N 7.48%; experimental: C 49.60%, H 5.21%, N 7.43%.

##### 4.1.2.6 Synthesis of **4** (Bis­(1-(anthracen-9-ylmethyl)-3-butylimidazol-2-ylidene) Gold Chloride)

**13** (726 mg, 2.07 × 10^–3^ mol) was dissolved in anhydrous *N*,*N*-dimethylformamide. A 1.0 M solution of lithium bis­(trimethylsilyl)­amide in THF (2.5 μL, 2.5 × 10^–3^ mol) was added to the mixture, which was stirred for 15 min. [AuCl­(SMe_2_)] (305 mg, 1.04 × 10^–3^ mol) was dissolved in anhydrous *N*,*N*-dimethylformamide and added to the solution drop by drop. After 4 h, the solution was concentrated and diethyl ether was added to obtain a yellow solid (573 mg, yield: 64%). ^1^H NMR (400 MHz, CDCl_3_) δ (ppm): 8.50 (s, 2H, Ant H8), 8.26 (m, 4H, Ant H6–H10), 8.00 (m, 4H, Ant H3–H13), 7.54 – 7.42 (m, 8H, Ant H5–H11–H4-H12), 7.07 (d, 2H, *J* = 1.9 Hz), 6.73 (d,2H, *J* = 1.9 Hz), 6.25 (s, 4H, AntC**H**_**2**_Im), 4.15 (t, *J* = 7.2 Hz, 4H, NC**H**_**2**_CH_2_CH_2_CH_3_), 1.80 (m, 4H, NCH_2_C**H**_**2**_CH_2_CH_3_), 1.29 (m, 4H, NCH_2_CH_2_C**H**_**2**_CH_3_), 0.84 (t, *J* = 7.4 Hz, 6H, NCH_2_CH_2_CH_2_C**H**_**3**_). ^13^CNMR (400 MHz, CDCl_3_) δ (ppm): 182.92 (N**C**N), 131.53 (Ant **C7**), 131.15 (Ant **C2**), 130.08 (Ant **C8**), 129.68 (Ant **C3/C6**), 127.80 (Ant **C6/C3**), 125.54 (Ant **C4/C5**), 124.24 (Ant **C1**), 123.14 (Ant **C5/C4**), 121.96 (Im), 121.24 (Im), 51.69 (Ant**C**H_2_Im), 47.17 (N**C**H_2_CH_2_CH_2_CH_3_), 33.54 (NCH_2_ **C**H_2_CH_2_CH_3_), 19.87 (NCH_2_CH_2_ **C**H_2_CH_3_), 13.76 (NCH_2_CH_2_CH_2_ **C**H_3_). Elemental analysis for C_44_H_44_AuN_4_Cl calculated: C 61.37%, H 5.15%, N 6.51%; experimental: C 61.28%, H 5.13%, N 6.48%.

#### 4.1.3 In Solution Behavior

##### 4.1.3.1 Water Solubility and Stability

The various complexes were dissolved until saturation in 600 μL of a 6.94 mM solution of DMSO_2_ in D_2_O. ^1^H NMR spectra were registered and the solubility values were calculated referring to the DMSO_2_ peak. The stability of the gold complexes was evaluated by dissolving the compounds in DMSO ([**3**] = 10^–4^ M, [**4**] = 6 × 10^–3^ M, [**5**] = 10^–5^ M) and recording UV–vis absorption spectra of the solutions every 30 min for 48 h at 25 °C. The stability in PBS buffer was evaluated by adding solutions of the complexes in DMSO to the buffer to obtain a 5 × 10^–5^ M solution. UV–vis absorption spectra of the solutions were registered every 30 min for 48 h at 25 °C.

##### 4.1.3.2 Log *P* Determination

Water (500 mL, distilled after Milli-Q purification) and *n*-octanol (500 mL) were shaken together for 72 h to allow saturation of both phases and kept resting for 1 week to allow the full separation of the two phases. A few milligrams of each complex were dissolved in *n*-octanol and an equal volume of water was added. The mixture was mixed for 10 min and centrifuged at 3000 rpm for 6 min to allow separation. UV–vis absorption spectra of both the aqueous organic phases were registered. Log *P* was calculated as Log *P* (Log *P* = log­(Absorption_octanol_/Absorption_water_)).

##### 4.1.3.3 UV–Vis Interaction Experiments with HSA

Stock solutions of each gold NHC complex in DMSO were prepared (10^–2^ M). Aliquots of each stock solution were mixed with a few μL of a stock solution of HAS in ultrapure water (10^–3^ M) at a 1:3 and 1:1 protein-to-metal ratio and diluted with PBS buffer. UV–vis spectra of the solutions were recorded every 30 min for 24/48 h at 37 °C.

##### 4.1.3.4 ESI-MS Interaction Experiments with HSA

Adducts were prepared by incubating HSA directly with the different metallodrugs at a 1:1 protein-to-metal ratio. The mixtures were incubated in ultrapure water for 24 h at 37 °C.

The protein solutions were further diluted with water to a final protein concentration of 10^–6^ M. Just before analysis, 0.1% (v/v) LC-MS grade formic acid was added. The ESI mass spectra were acquired through direct injection at a flow rate of 7 μL/min in a TripleTOF 5600^+^ high-resolution mass spectrometer (Sciex, Framingham, MA, U.S.A.), equipped with a DuoSpray interface operating with an ESI probe. The ESI source parameters were set as follows for all samples. Positive polarity, Ionspray Voltage Floating 5500 V, Temperature 0, Ion source Gas 1 (GS1) 40 L/min; Ion source Gas 2 (GS2) 0 L/min; Curtain Gas (CUR) 20 L/min, Declustering Potential (DP) 200 V, Collision Energy (CE) 10 V, acquisition range 1000–2600 *m*/*z*.

#### 4.1.4 Single-Crystal X-ray Diffraction

Single-crystal X-ray diffraction (SCXRD) was performed with a Bruker D8 Venture instrument equipped with a microfocus Mo source (Kα radiation, λ = 0.71073 Å) and a 2D Photon III detector.

The CCDC code for the structure is 2355406. The main experimental details regarding the determination of the structure of (**4**) by SCXRD are reported in Table S1. The unit cell was identified and initially refined using APEX4.^52^ Successively, data were integrated and reduced using SAINT^53^ and XPREP.^54^ Absorption effects were corrected using SADABS.^55^ The structure was solved and refined with the aid of SHELXL.^56^ All hydrogen atoms were fixed at the calculated position.

Starting from the experimentally determined X-ray structure, a geometry refinement was performed to optimize the C–H bond lengths using the MMFF94 (Merck Molecular Force Field) as implemented in *Spartan’20* software.^57^ Density functional theory (DFT) calculations were subsequently carried out using the B3LYP functional with Grimme’s D3 dispersion correction and Becke–Johnson damping (B3LYP-D3­(BJ)).^58^ The def2-SVP basis set was applied for all main group elements (C, H, N, Cl), while for gold, the Stuttgart-Dresden (SDD) basis set with the corresponding MWB60 effective core potential was employed. All DFT computations were performed using the *Gaussian 16* software package (Revision C.01).^59^ The electron density and related surface analyses were conducted with the *Multiwfn* software, version 3.8.^60–62^ Hirshfeld surfaces^63^ and fingerprint plots^64^ were calculated using *CrystalExplorer 17* program^65^ for the compound (**4**) to better understand the intermolecular interactions inside the crystal lattice. This study was performed starting from the structural parameters of (**4**) and the graphs of Hirshfeld surfaces were mapped over *d*_norm_ in the range of −0.1670 to 1.4140 arbitrary units (*d*_norm_ is the normalized distance from a given point to the nearest external (*d*_e_), internal (*d*_i_) atom and van der Waals radius). The fingerprint plots were obtained with respect to *d*_e_ and *d*_i_ distances, which provide a description of the intermolecular contacts and allow a quantification of specific interactions.^66^

### 4.2 Biology

#### 4.2.1 General Remarks

Thiazolyl blue tetrazolium bromide (MTT) was obtained from Merck Life Science (Milan, Italy). A2780 human ovarian cancer cell line was purchased from Creative Bioarray (NY 11967, USA): A2780 sensitive to cisplatin (Lot N° CSC-C9491J) and A2780 resistant to cisplatin (Lot N° CSC-C9492J); cell lines were maintained in RPMI-1640 supplemented with 10% FBS and antibiotics (penicillin, 100 U/mL; streptomycin, 100 μg/mL) at 37 °C in a 5% CO_2_ humidified atmosphere and subcultured twice weekly. Split 1:5 (3–6 × 10^4^ cells per mL). Cell lines have been routinely tested for mycoplasma (Lonza’s MycoAlert Mycoplasma Detection Assays).

#### 4.2.2 MTT Cytotoxicity Assay

The cytotoxic effects of the complexes were determined using the colorimetric MTT [3-(4,5-dimethylthiazol-2-yl)-2,5-diphenyltetrazolium bromide] assay. Viable cells metabolize MTT into formazan, a purple-colored compound detectable at 595 nm. Exponentially growing cells were seeded in 96-well plates at a density of 10^4^ cells/well. After 24 h, cells were treated with increasing concentrations of the metal compounds (ranging from 100 nM to 100 μM), with each concentration tested in triplicate. Following a 72 h incubation, 0.5 mg/mL of MTT was added to each well and incubated at 37 °C for 1 h. The resulting formazan crystals were dissolved in 100 μL of DMSO, and the optical density was measured using a BioTek Synergy H1 (Agilent) microplate reader. The half-maximal inhibitory concentration (IC_50_) for each compound was calculated from the absorbance data using GraphPad Prism software (version 6.0; GraphPad Software, San Diego, CA, USA). Each MTT assay was performed at least in triplicate, and the results are reported in Table 2 as IC_50_ mean values ± standard deviation (SD).

#### 4.2.3 Uptake Analysis Using Flow Cytometry

Cells (4 × 10^5^) were seeded in p60 culture plates. After 24 h, the cells were detached, resuspended in PBS, and labeled with the fluorescent compounds (10 μM) for 10 min at 37 °C. Subsequently, cells were washed in PBS and resuspended in 500 μL of PBS. Fluorescent probe intensity was measured using BD Fluorescence-activated cell sorting (FACS) Canto II (BD Biosciences, Becton Dickinson Europe Holdings SAS—Francia). Particularly, median fluorescence intensity (MFI) was measured in the Pacific Blue channel (421 nm) and normalized on the Forward Scatter (FSC-A) parameter, an indicator of cell size, to take into account the difference in size between cisplatin-sensitive and cisplatin-resistant ovarian cell lines.

#### 4.2.4 Subcellular Fractionation and Analysis of Intracellular Compound Accumulation

Subcellular fractionation was performed according to a previously published protocol with modifications.^67^ Cells (2 × 10^6^) were seeded in p100 culture plates. After 24 h, the cells were washed twice with ice-cold phosphate-buffered saline (PBS) and resuspended in 500 μL isotonic fractionation buffer (250 mM sucrose, 20 mM Hepes, 10 mM KCl, 2 mM MgCl_2_, EDTA 1 mM, EGTA 1 mM) supplemented with 1 mM DTT and 0.1% protease inhibitor cocktails. Cells were mechanically disrupted by passing through a 1 mL syringe with a 27 gauge needle (10 times) and then incubated in ice for 20 min. The resulting lysate was centrifuged at 1,000*g* for 10 min at 4 °C. The pellet, containing cell debris and nuclei, was discarded. The supernatant was collected into a fresh tube and further centrifuged at 10,000*g* for 20 min at 4 °C to obtain the mitochondrial fraction (pellet) and the cytosolic fraction (supernatant). The mitochondrial pellet was washed once with fractionation buffer to minimize cross-contamination and resuspended in an appropriate volume to be analyzed in the flow cytometer. To quantify the fluorescent intensity of complexes **3** and **4** in the cytosolic and mitochondrial fractions, an equal volume of these two fractions was analyzed using a microplate reader (Biotek Sinergy H1, Agilent Technologies). Fluorescence intensity was normalized to the protein concentration determined in the cytosolic and mitochondrial fractions.

#### 4.2.5 Western Blot Analysis

Mitochondria pellet was lysed in ice-cold RIPA buffer (150 mM NaCl, 100 mM NaF, 2 mM EGTA, 50 mM Tris-HCl pH 7.5, 5 mM orthovanadate, 1% Triton X-100, 0.1% SDS, and 0.1% protease/phosphatase inhibitor cocktails) for 10 min. Lysates were clarified by centrifugation at 14,000 rpm for 10 min. Protein concentration of mitochondrial lysate and cytosolic fraction was determined using the Bradford assay. Equal amounts of protein were then boiled for 5 min in 4× Laemmli buffer (0.5 M Tris-HCl pH 6.8, 10% SDS, 20% glycerol, β-mercaptoethanol, and 0.1% bromophenol blue). Proteins were separated by 12% SDS-PAGE and transferred onto PVDF membranes using the Trans-Blot Turbo Transfer System (Bio-Rad). Membranes were blocked and probed with primary antibodies against GAPDH (1:1000 in 2% milk; Santa Cruz Biotechnology) and VDAC (1:2000 in 2% milk; 11663–1-AP Proteintech) overnight at 4 °C. Following incubation with HRP-conjugated antimouse or antirabbit IgG secondary antibodies (1:5000; Santa Cruz Biotechnology), immunoreactive bands were detected using an ECL system (GE Healthcare) and imaged with the Amersham Imager 600. The blot was subjected to densitometric analysis using the ImageJ 1.53 program. The intensity of the immunostained bands was normalized with the total protein intensities measured by Coomassie brilliant blue R-250 from the same PVDF.

#### 4.2.6 Statistical Analysis

All the biological experiments were performed in triplicate. The statistical analysis was carried out by One-way ANOVA followed by Tukey’s multiple comparison test using GraphPad Prism 6. Data are presented as means ± standard deviation (SD). *p* < 0.05 were considered statistically significant (*p < 0.05; **p < 0.01; ***p < 0.001; ****p < 0.0001).

## Supplementary Material



## Notes

NMR characterization spectra, crystallographic data for compound **3**, water solubility studies, stability and HSA interaction studies, and emission of compound **5** in the FACS channels (PDF)

## ⊥

Department of Biotechnology, Chemistry and Pharmacy, University of Siena, Via Aldo Moro, 2, 53100 Siena, Italy
