# Supplementary material for: Fluorescent NHC Gold(I) Complexes as Tools for Target Identification and Metallomic Studies in Cancer Cells
Source: Inorg Chem. 2026 Aug 3;65(32):18593–607. doi: 10.1021/acs.inorgchem.6c01796 (PMC13488405; doi:10.1021/acs.inorgchem.6c01796)
Supplement: Supplementary file 1 [file ic6c01796_si_001.pdf]

Supporting information

Fluorescent NHC Gold(I) Complexes as Tools for Target Identification and Metallomic Studies in Cancer Cells

Ester Giorgi,[1,†] Matteo Boldrini,[1,†,‡] Francesca Binacchi,[1] Damiano Cirri,[1] Michele Mannelli,[2] Tania Gamberi,[2] Massimo Guelfi,[1] Matteo Becatti,[2] Lara Massai,[3] Chiara Gabbiani,[1] Alessandro Pratesi,[1,]* Luigi Messori[3,]*

[1] *Department of Chemistry and Industrial Chemistry, University of Pisa, Via G. Moruzzi 13, 56124 Pisa, Italy.*

[2] *Department of Experimental and Clinical Biomedical Sciences "Mario Serio", University of Florence, Viale G.B. Morgagni 50, 50134 Firenze, Italy.*

[3] *Department of Chemistry "U. Schiff", University of Florence, Via della Lastruccia 3–13, 50019 Sesto Fiorentino, Italy.*

* Corresponding authors: alessandro.pratesi@unipi.it (AP); luigi.messori@unifi.it (LM)

† These authors equally contributed to the work.

‡ Present address: Department of Biotechnology, Chemistry and Pharmacy, University of Siena, Via Aldo Moro, 2, 53100 Siena, Italy.

Table of Contents

NMR characterization spectra 2

Crystallographic data for compound **3** 7

Water solubility studies 12

Stability and HSA interaction studies 13

Emission of compound **5** in the FACS channels 16

NMR characterization spectra

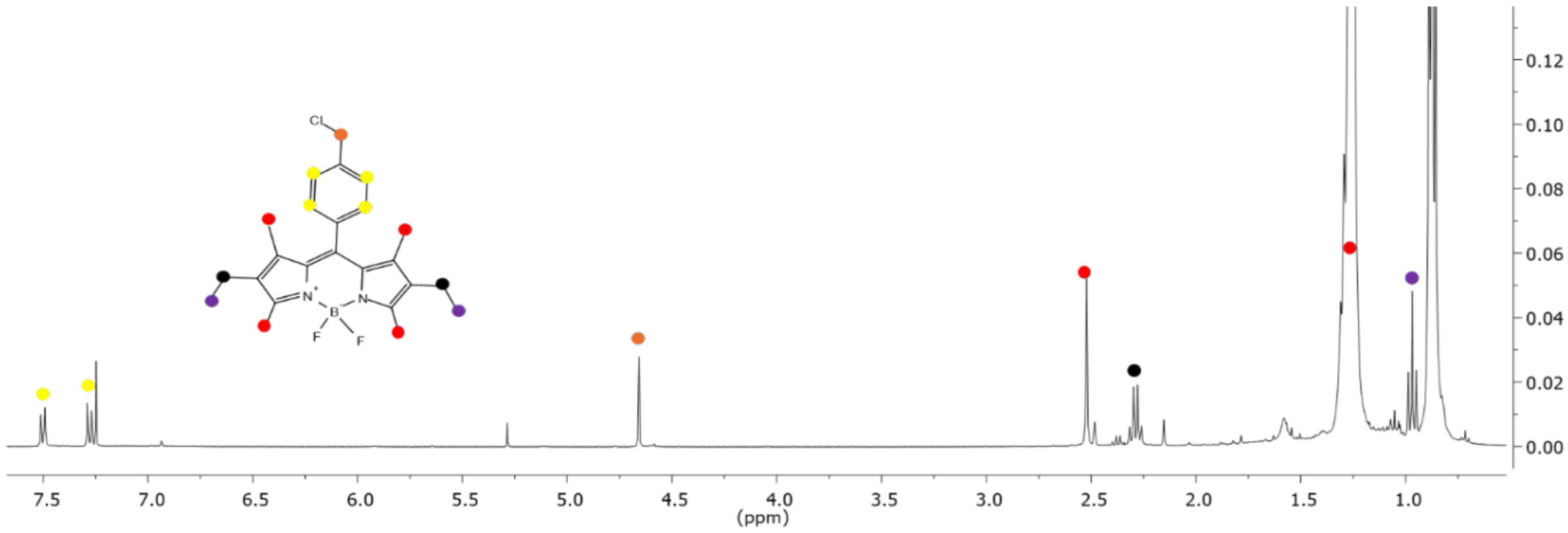

Figure S1 ^{1}H NMR spectrum of **10** in $CDCl_3$.

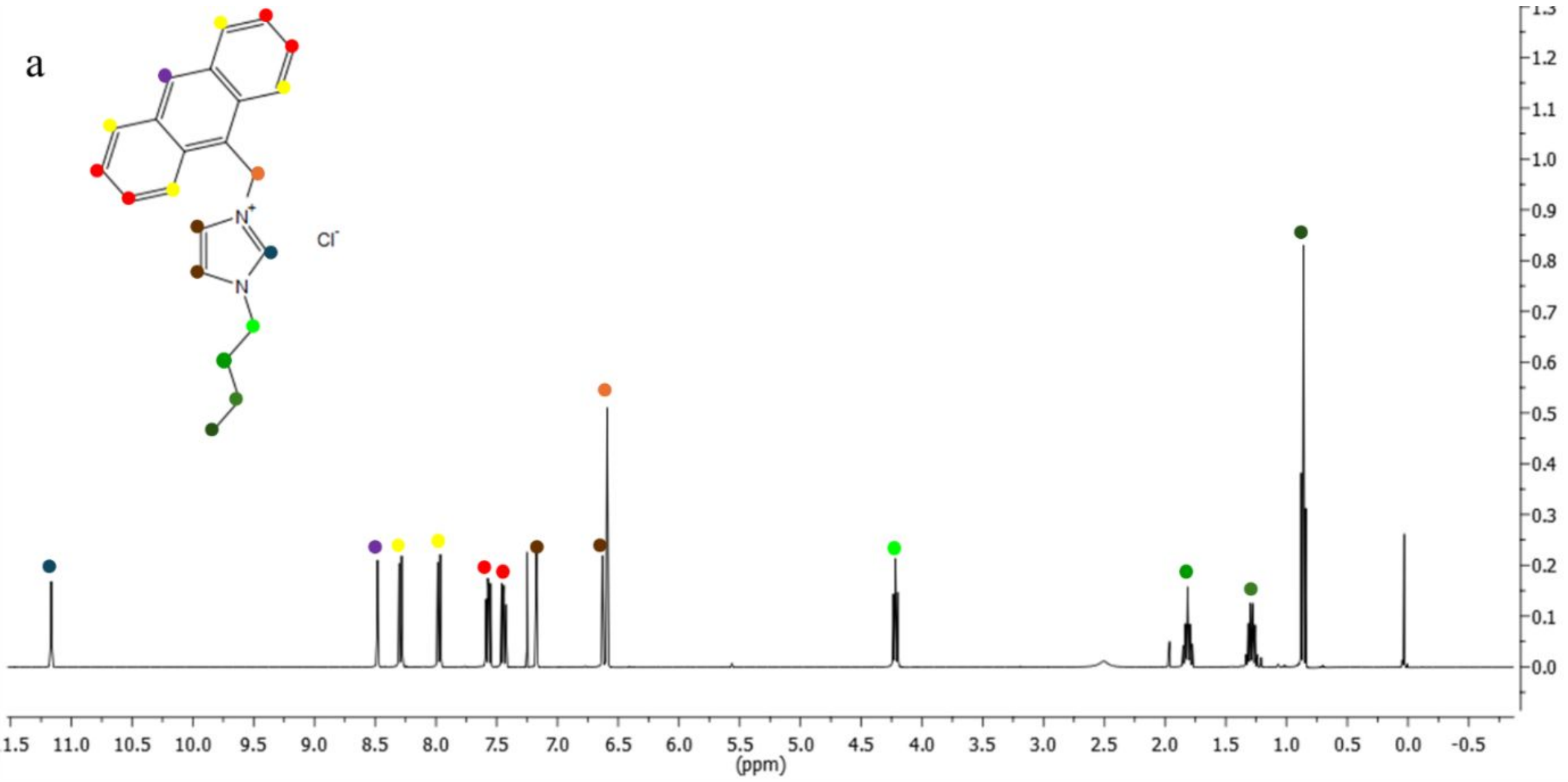

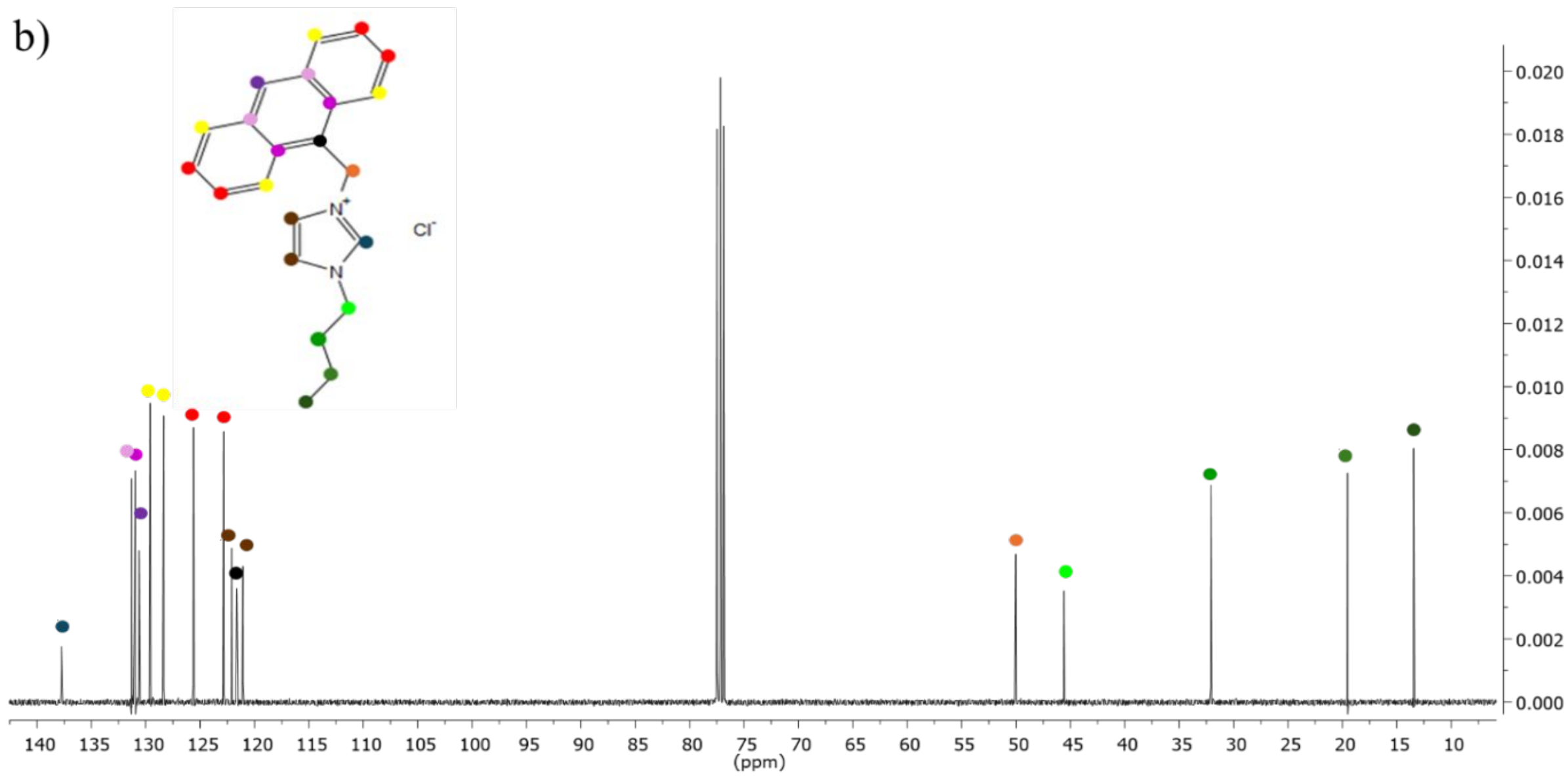

Figure S2 ^{1}H (a) and ^{13}C (b) NMR spectra of **13** in $CDCl_3$.

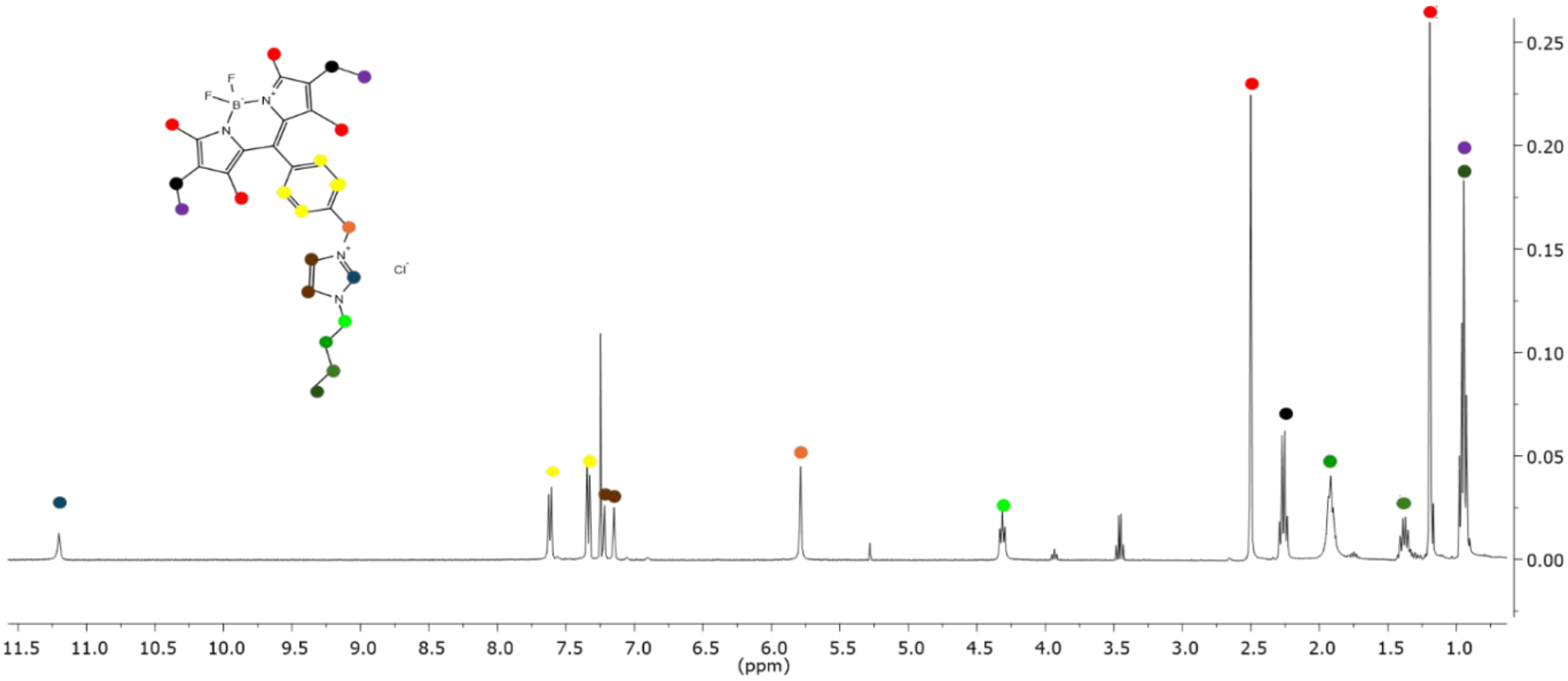

Figure S3 ^{1}H NMR spectrum of **14** in $CDCl_3$.

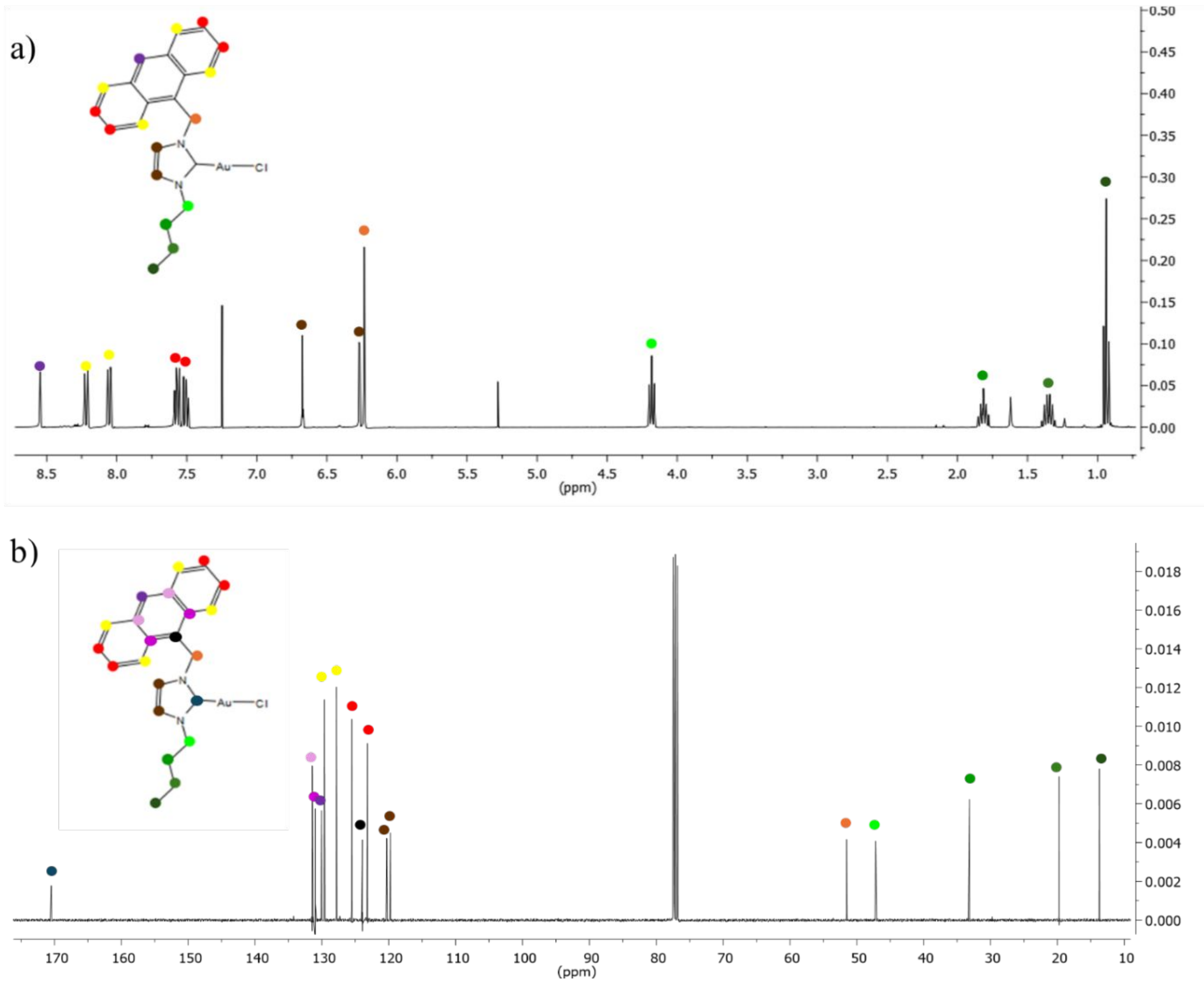

Figure S4 ^{1}H (a) and ^{13}C (b) NMR spectra of **3** in $CDCl_3$.

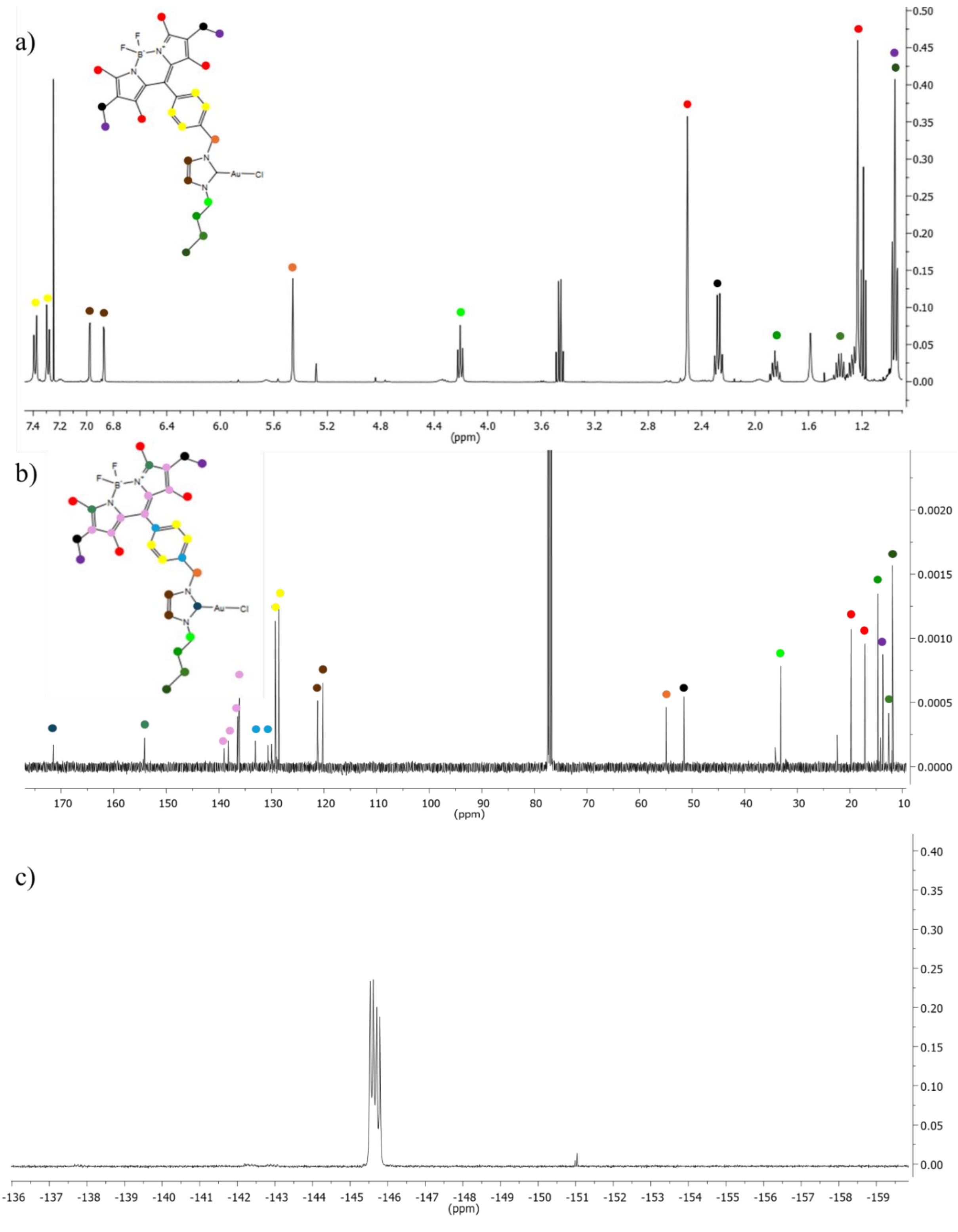

Figure S5 ^{1}H (a), ^{13}C (b) and ^{19}F (c) NMR spectra of **5** in $CDCl_3$.

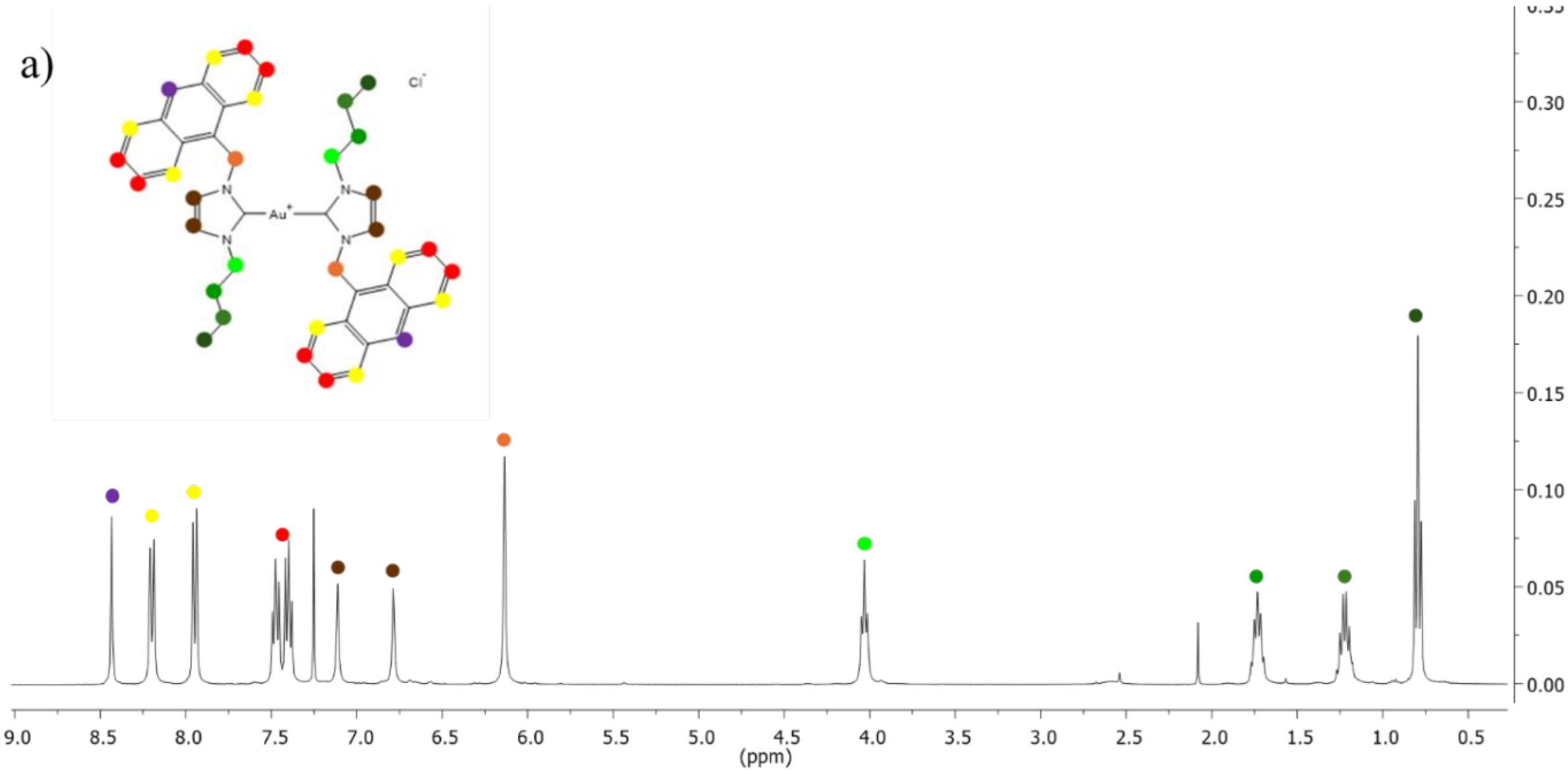

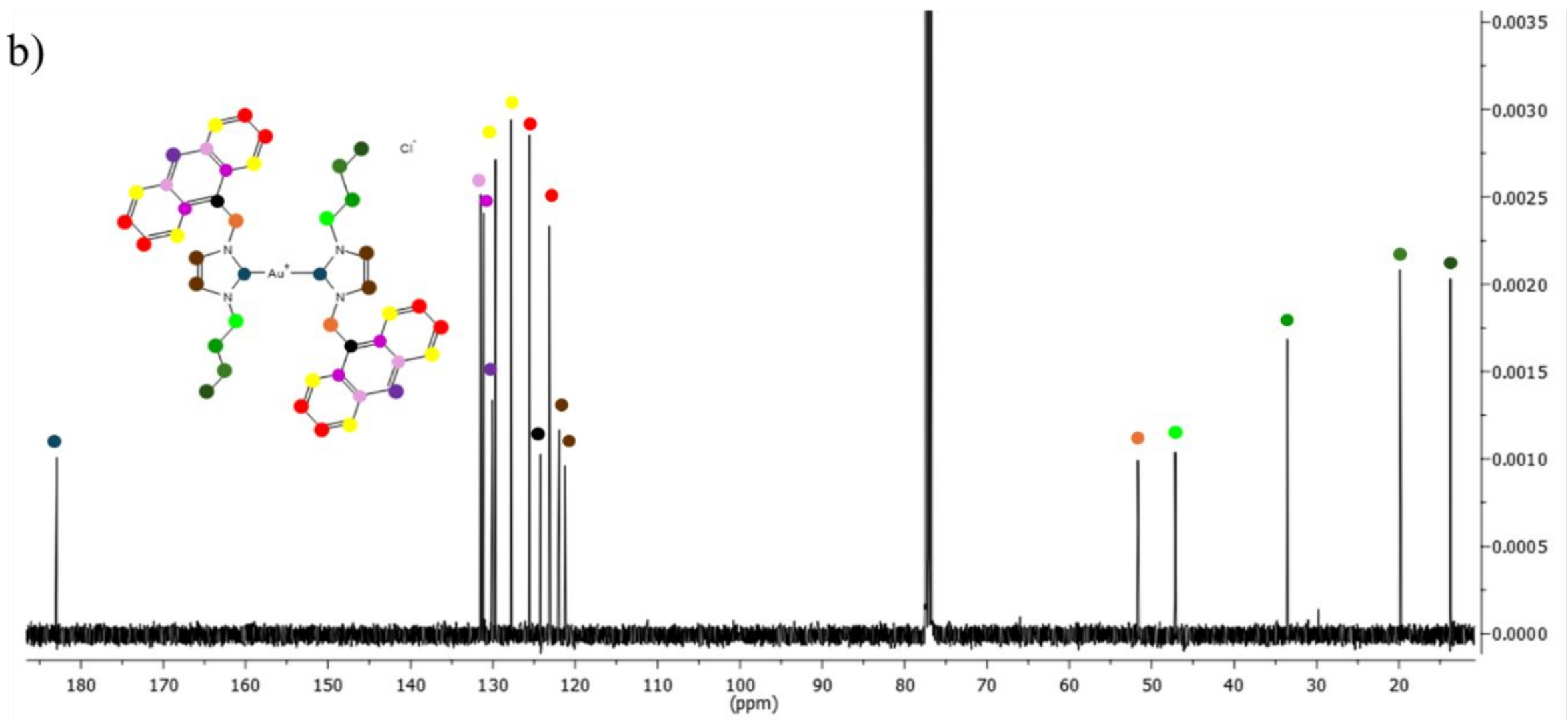

Figure S6 ^{1}H (a) and ^{13}C (b) NMR spectra of **4** in $CDCl_3$.

a)

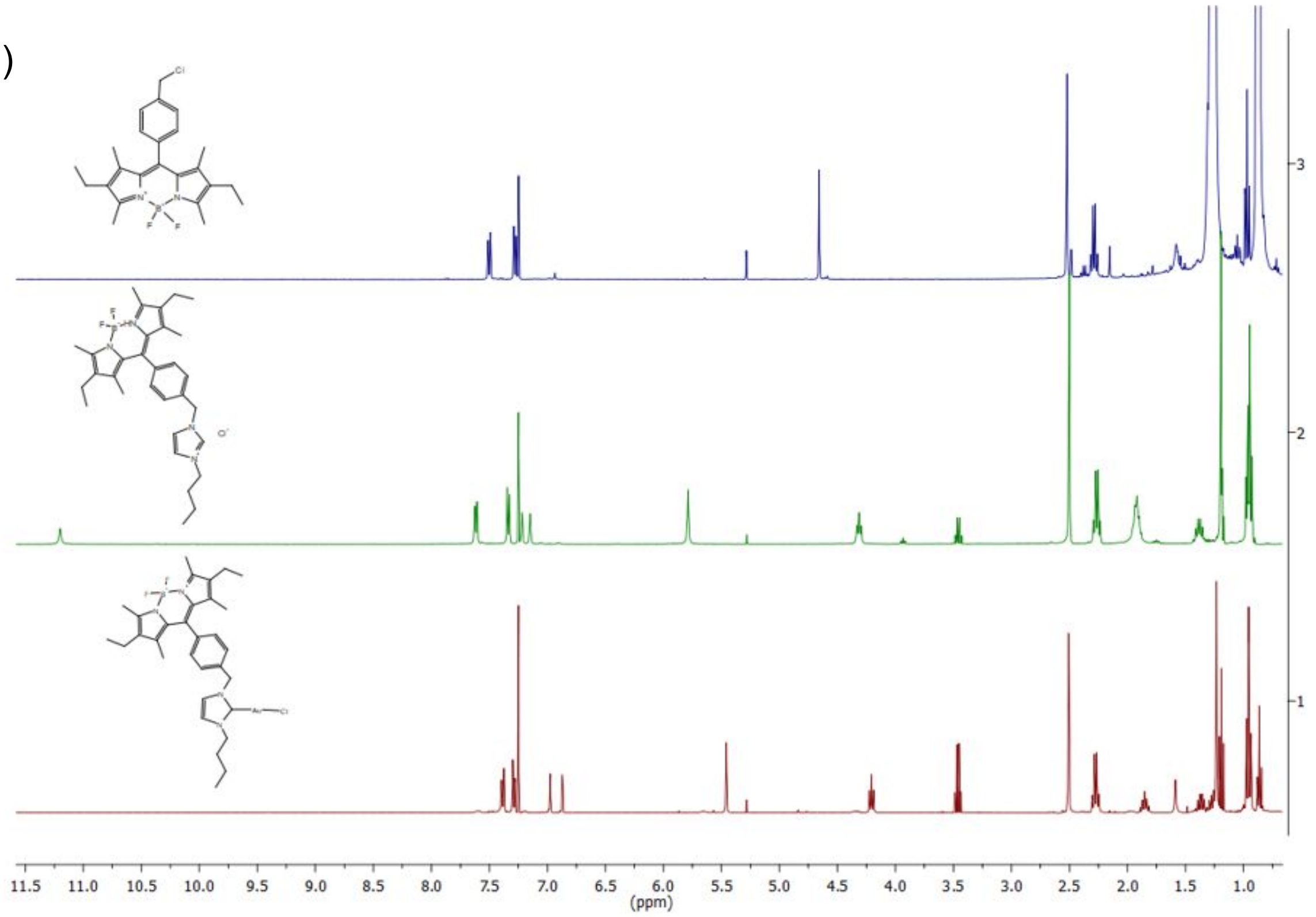

b)

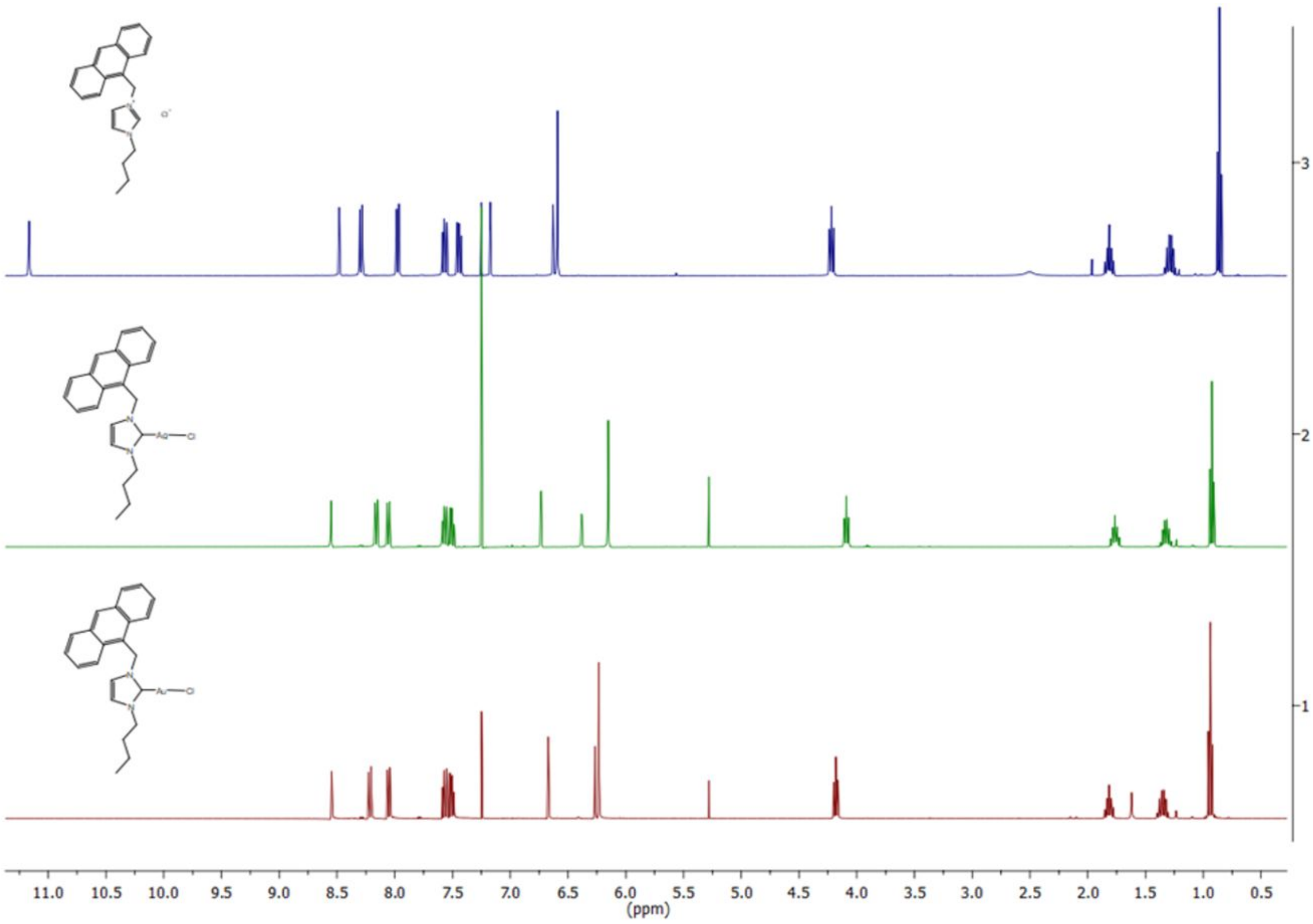

Figure S7 ^{1}H NMR spectra comparison of compounds **10**, **14** and **5** (a) and **13**, **15** and **3** (b) in $CDCl_3$.

Crystallographic data for compound 3

Table S1. Percentage distribution of main reciprocal contacts based on fingerprint plots and their distribution on the Hirshfeld surface.

Contact surface distribution	Contact type	Percentage
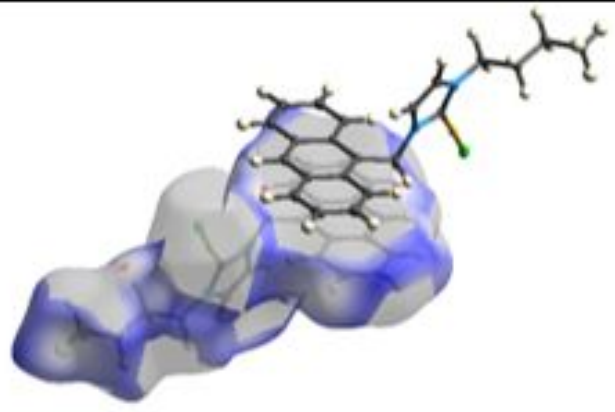	H…H	51.8 %
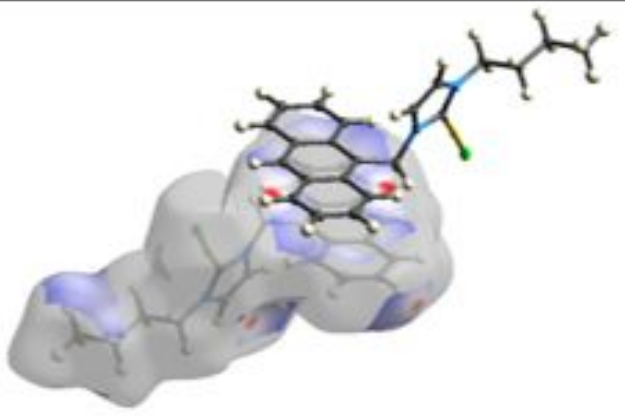	C…H/H…C	21.6 %
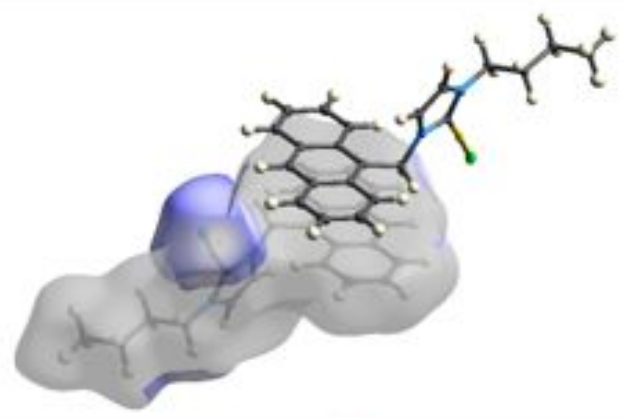	Cl…H/H…Cl	14.3 %
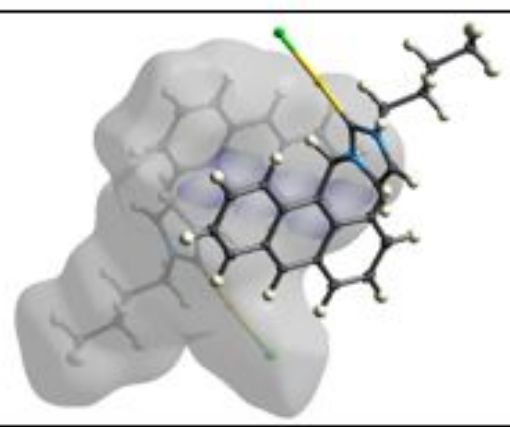	C…C	4.7 %
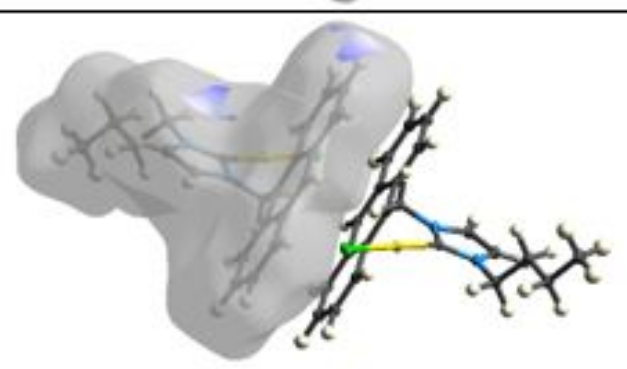	N…H/H…N	1.4 %

Table S2. Sample, crystal data, data collection and structure refinement for **3.**

CCDC ID	2355406
Formula	$C_{22}H_{22}AuClN_2$
FW, g mol^{-1}	546.83
T, K	100(2)
λ, Å	0.71073
Crystal system	Triclinic
Space group	*P*-1
a, Å	9.1416(3)
b, Å	10.6942(4)
c, Å	11.1580(4) Å
α,°	106.4060(10)
β, °	111.1850(10)
γ,°	94.9070(10)
Cell volume, Å^3	953.99(6)
Z	2
Density calculated, g cm^{-3}	1.904
Absorption coefficient, mm^{-1}	7.858
F(000)	528
Crystal size, mm	0.072 x 0.064 x 0.022
θ limits, °	2.03 to 33.76
Reflection collected	73237
Independent reflection	7632 [R_{int} = 0.0480]
Data/restraints/parameters	7632/0/236
Goodness of fit on F^2	1.126
R_1 ($I > 2\sigma(I)$)	0.0220
wR_2 ($I > 2\sigma(I)$)	0.0421
R_1 (all data)	0.0264
wR_2 (all data)	0.0432
Largest diff. peak and hole, e Å^{-3}	1.021 and −1.672

Table S3. Selected bond lengths (Å) and bond angles (°) of **3** involving Au, Cl, C16 and N atoms.

Au1-Cl1	2.2883(5)	Au1-C16	1.979(2)
C16-N1	1.356(3)	C16-N2	1.356(3)
C16-Au1-Cl1	177.09(6)	N1-C16-N2	104.68(18)
N1-C16-Au1	124.89(15)	N2-C16-Au1	130.42(16)

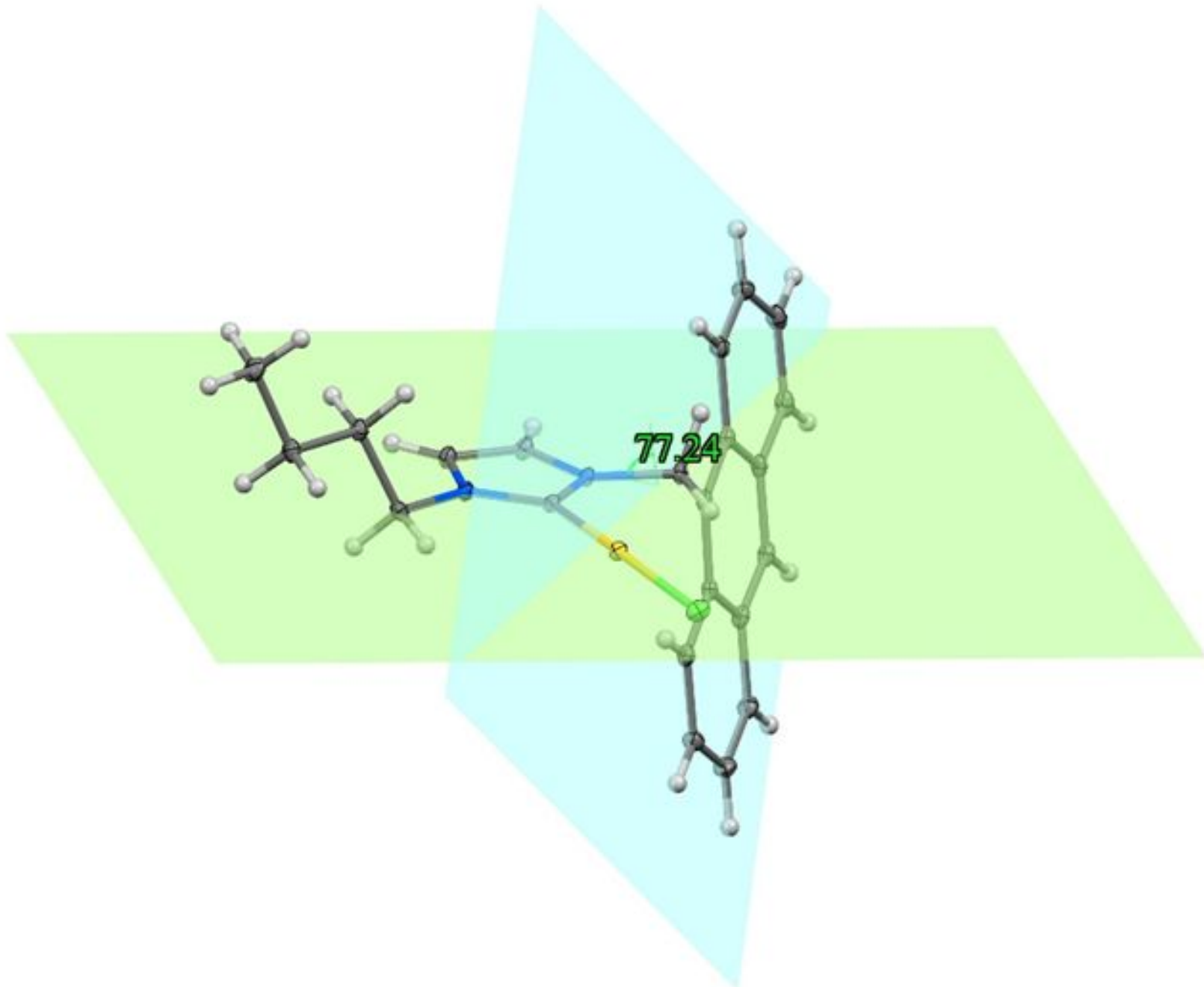

Figure S8. Angle between the plane of the anthracene fragment and that of the *N*-heteroaromatic carbene.

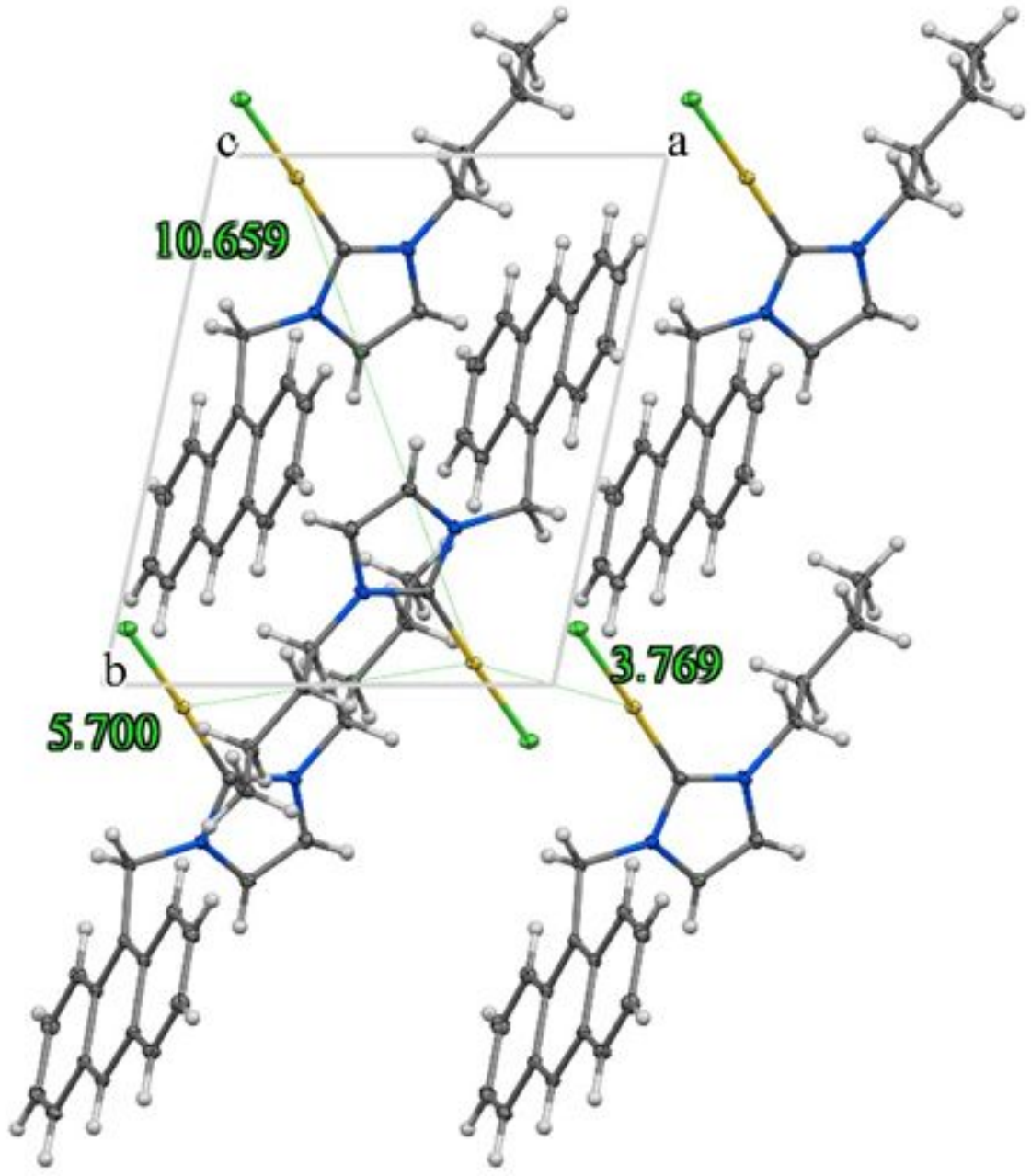

Figure S9. Main Au…Au distances inside the lattice.

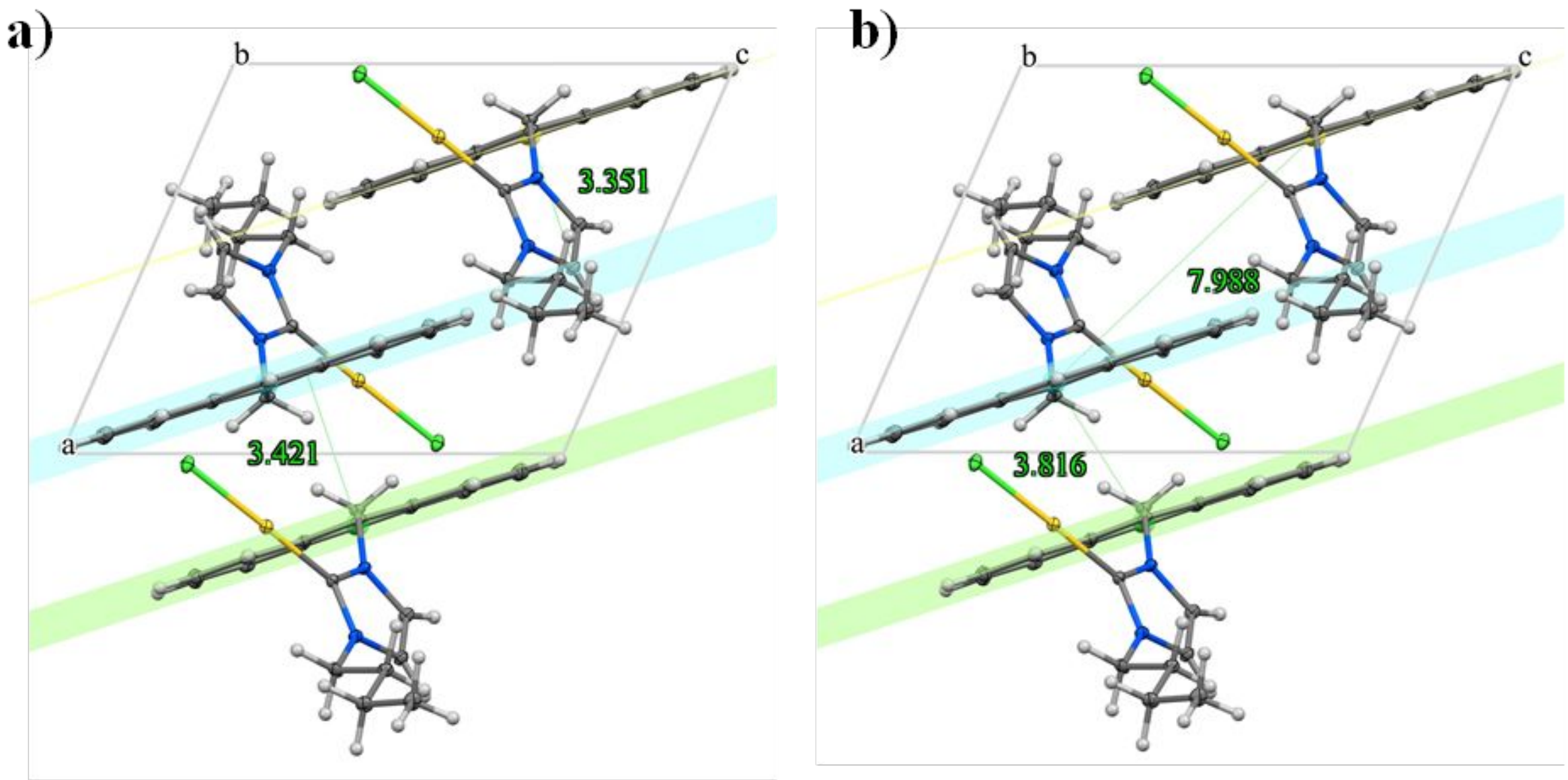

Figure S10. Distances between anthracenyl planes (a) between centroids and planes, (b) between centroids.

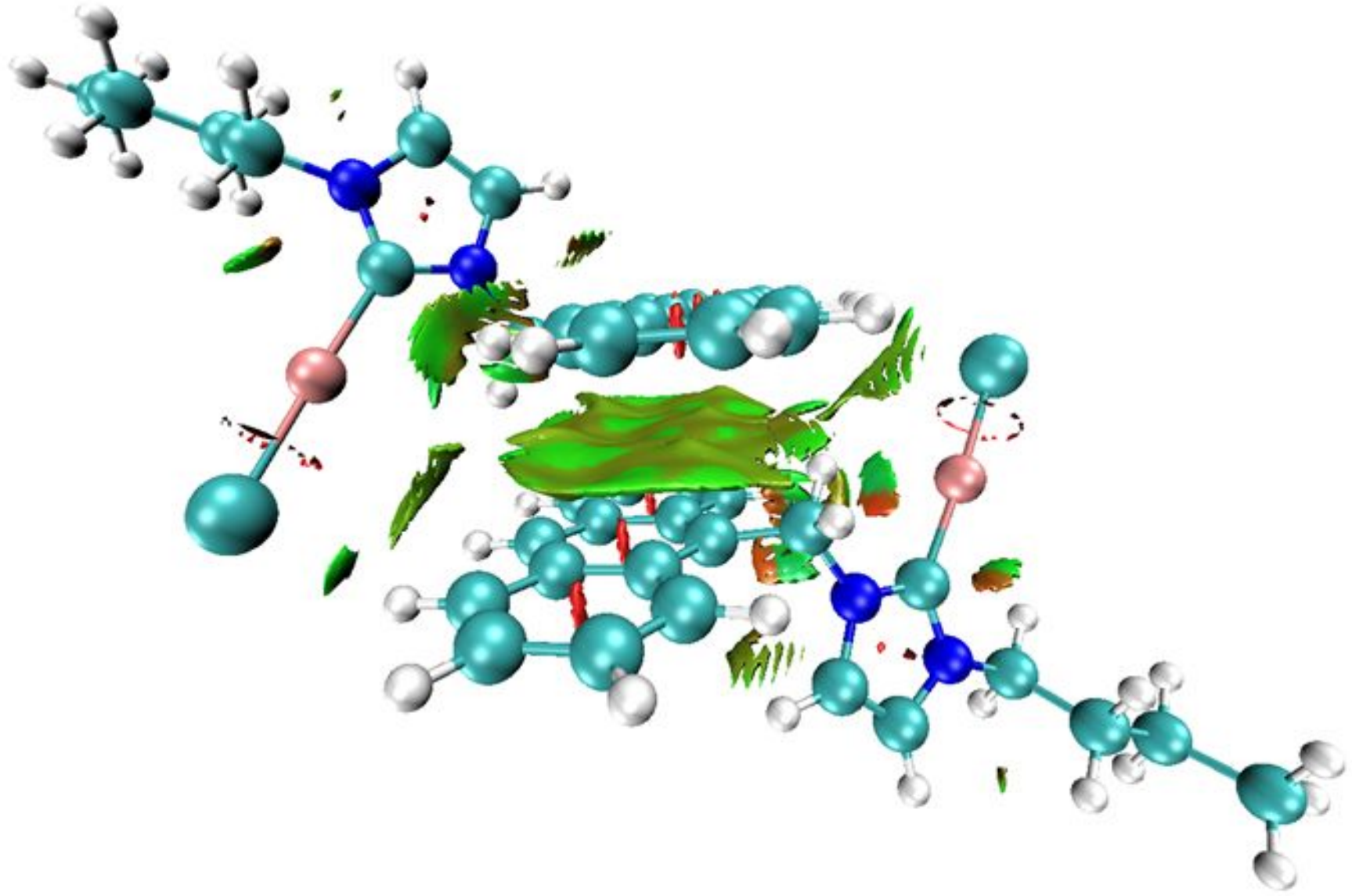

Figure S11. Non-covalent interaction (NCI) isosurface plot calculated with *Multiwfn* 3.8, highlighting weak intermolecular interactions between the extended aromatic systems of the two gold(I) complex units within the crystal structure. The isosurface is colored according to the sign(λ_2)ρ function: green regions correspond to weak attractive van der Waals interactions, while red regions indicate strong steric repulsion.

Water solubility studies

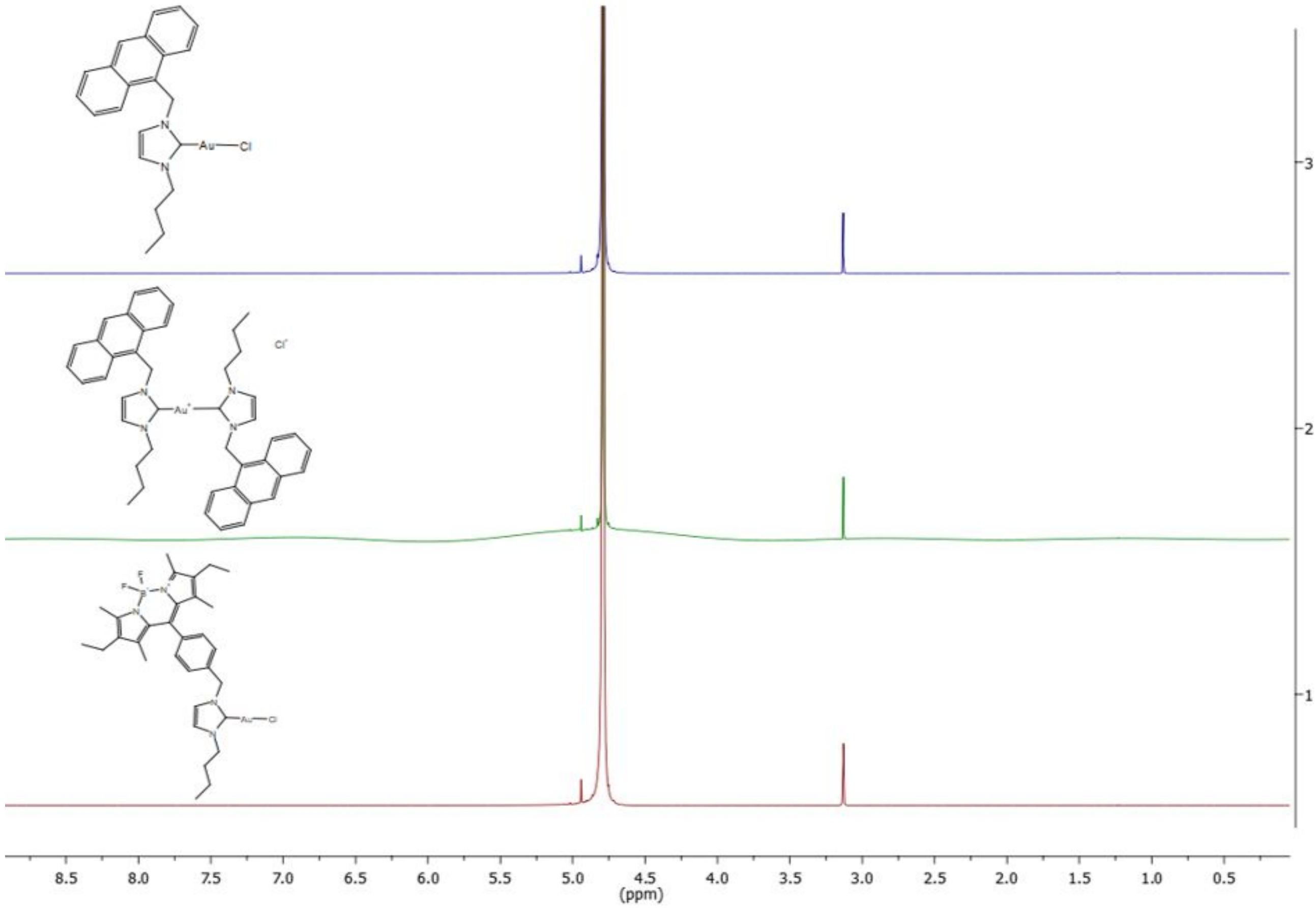

Figure S12. ^{1}H NMR spectra of the synthesized compounds in $DMSO_2/D_2O$ solution.

Stability and HSA interaction studies

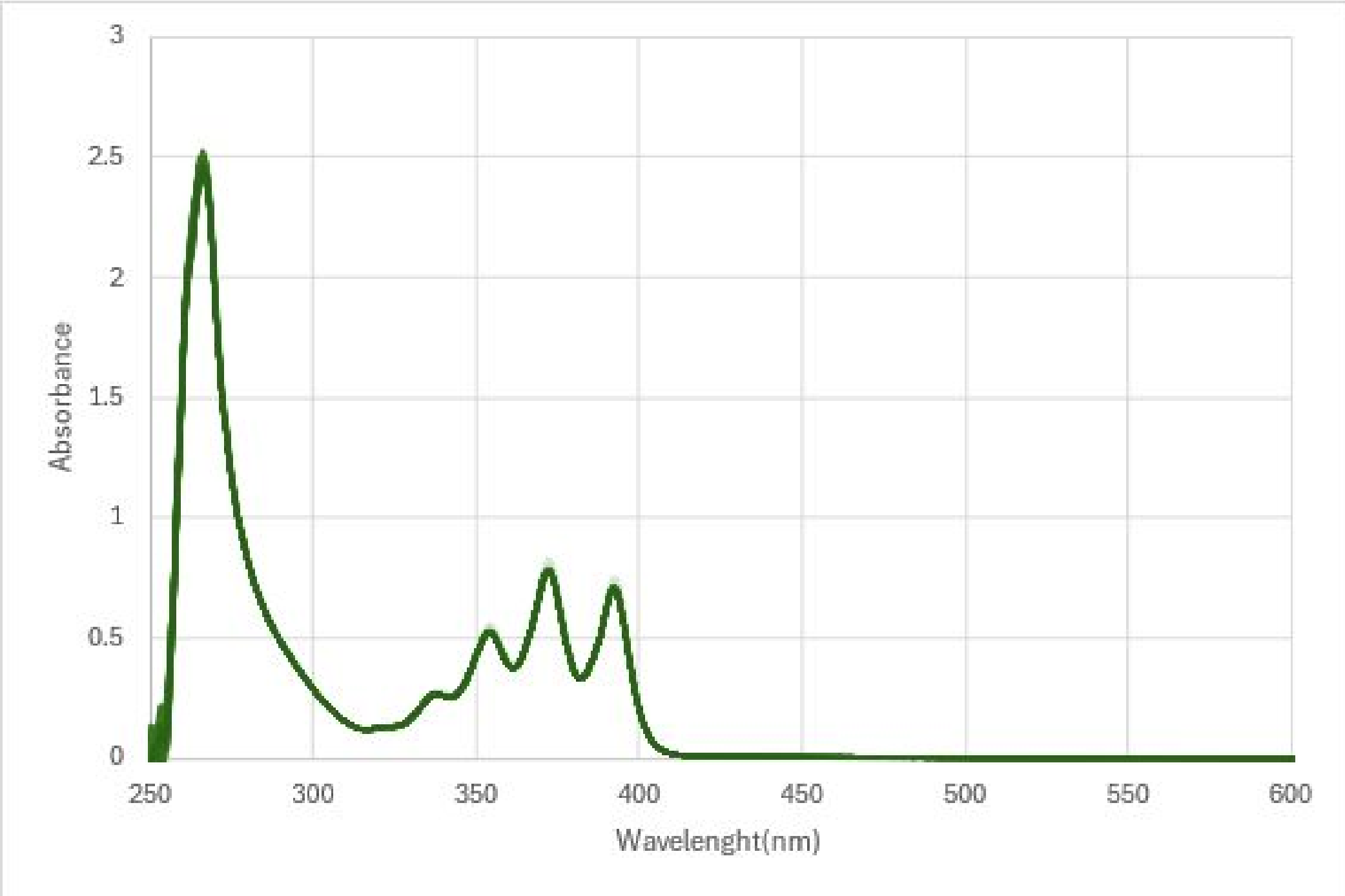

Figure S13. UV-Vis absorption spectra of **4** in DMSO at 25 °C over 48 h.

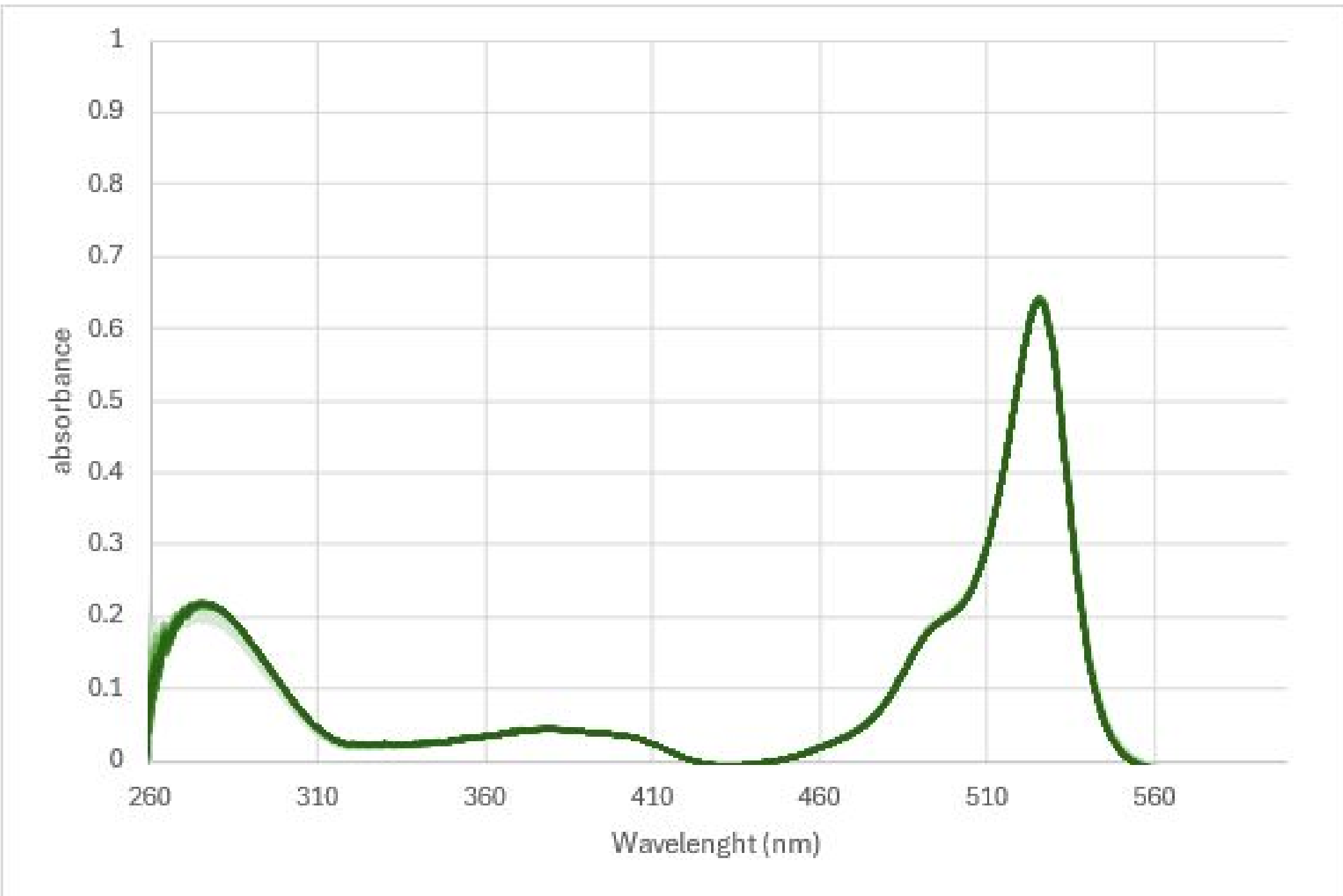

Figure S14. UV-Vis absorption spectra of **5** in DMSO at 25 °C over 48 h.

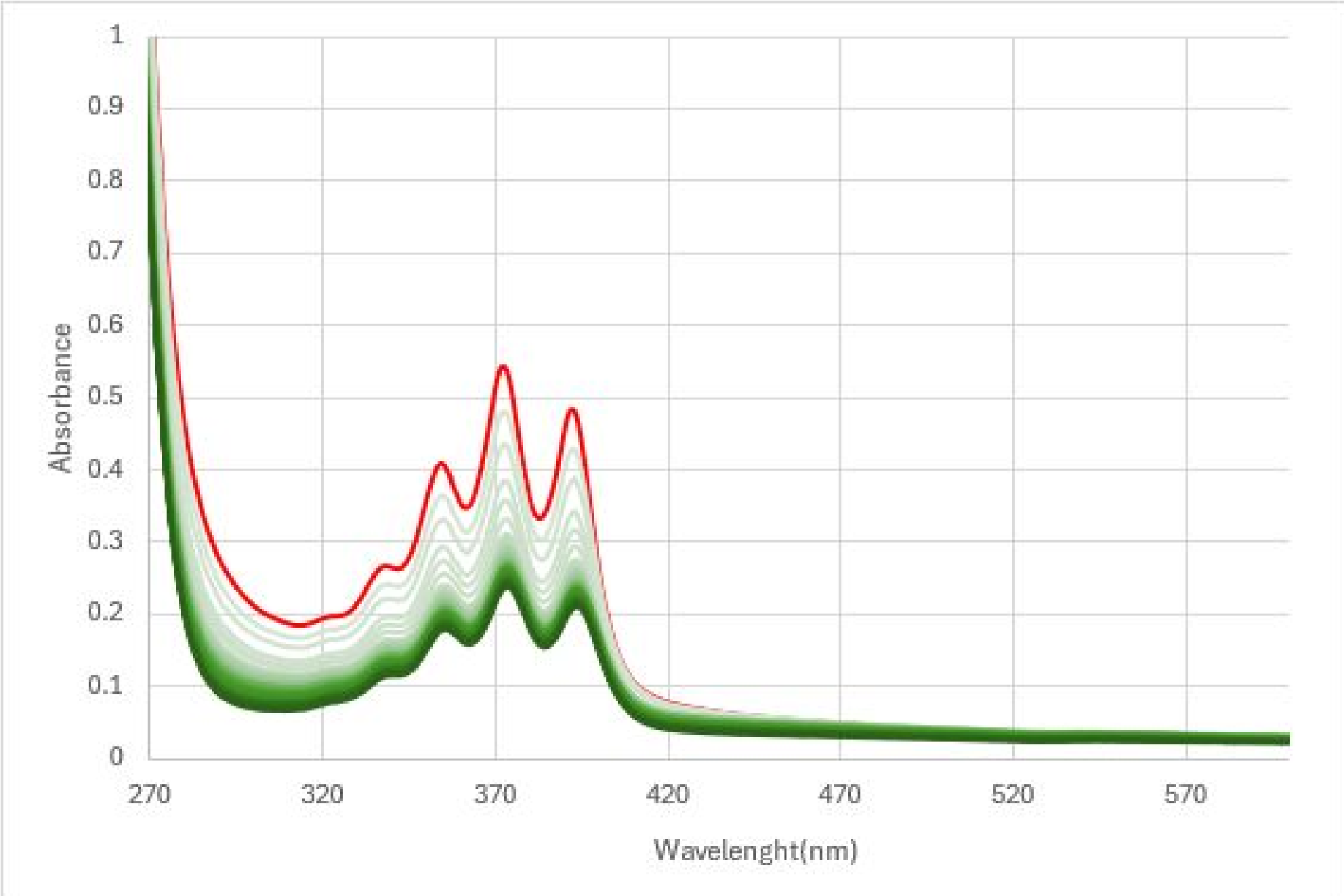

Figure S15. UV-Vis absorption spectra of **4** in PBS at 25 °C at t = 0 h (red) and over 48 h (green).

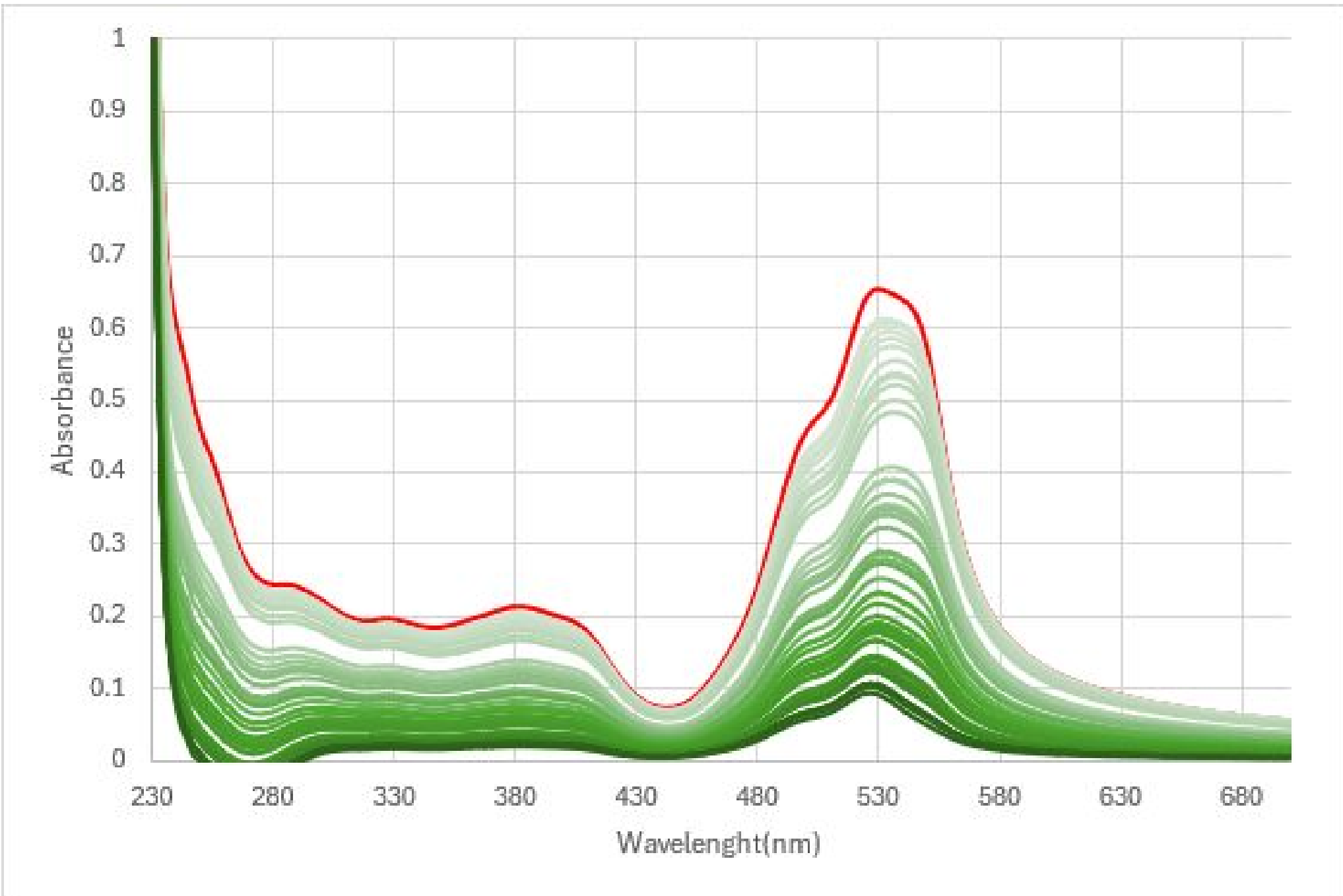

Figure S16. UV-Vis absorption spectra of **5** in PBS at 25 °C at t = 0 h (red) and over 48 h (green).

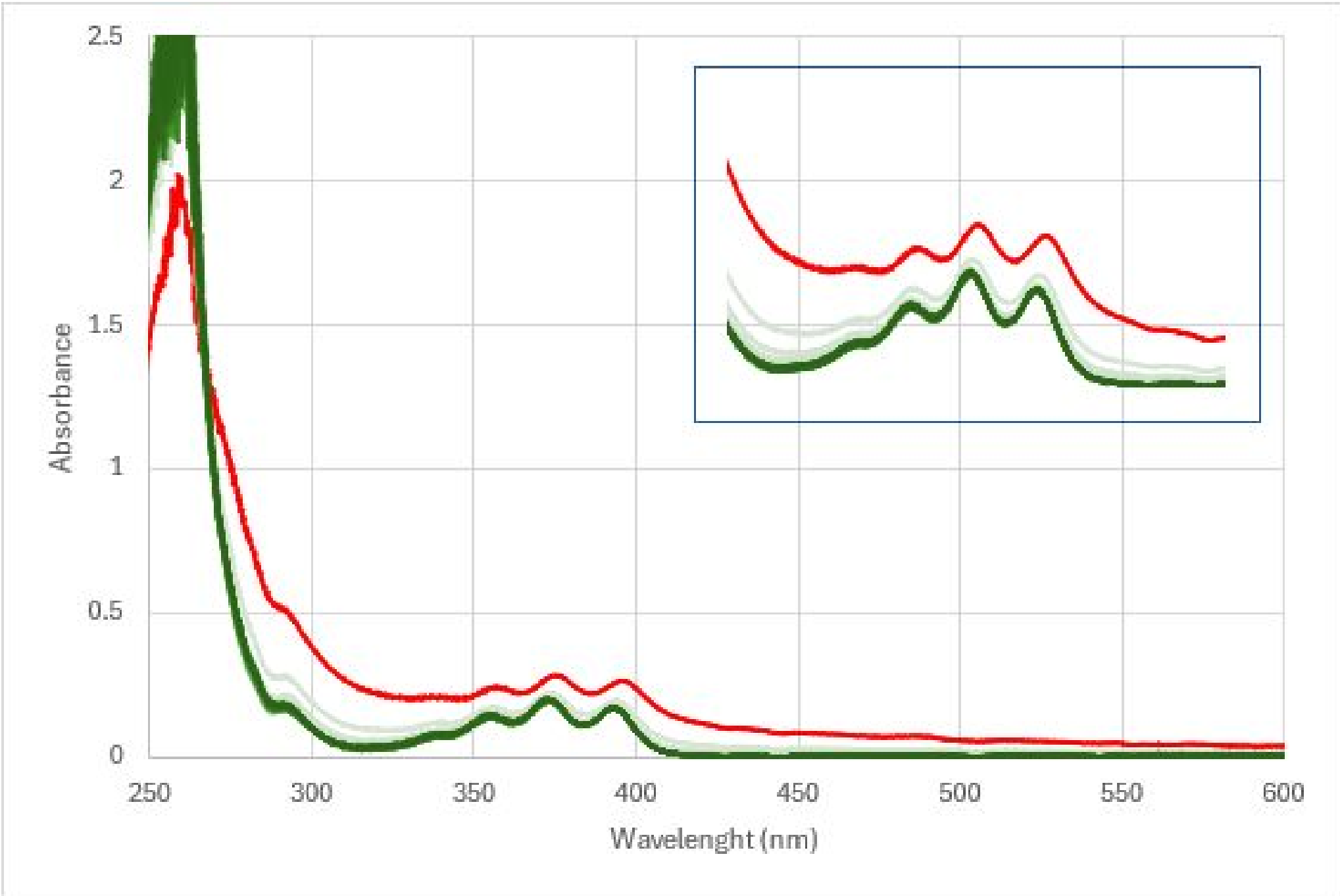

Figure S17. UV-Vis spectra of **3** incubated with HSA (1:1 metal to protein) in PBS at 37 °C at t = 0 h (red) and over 24 h (green).

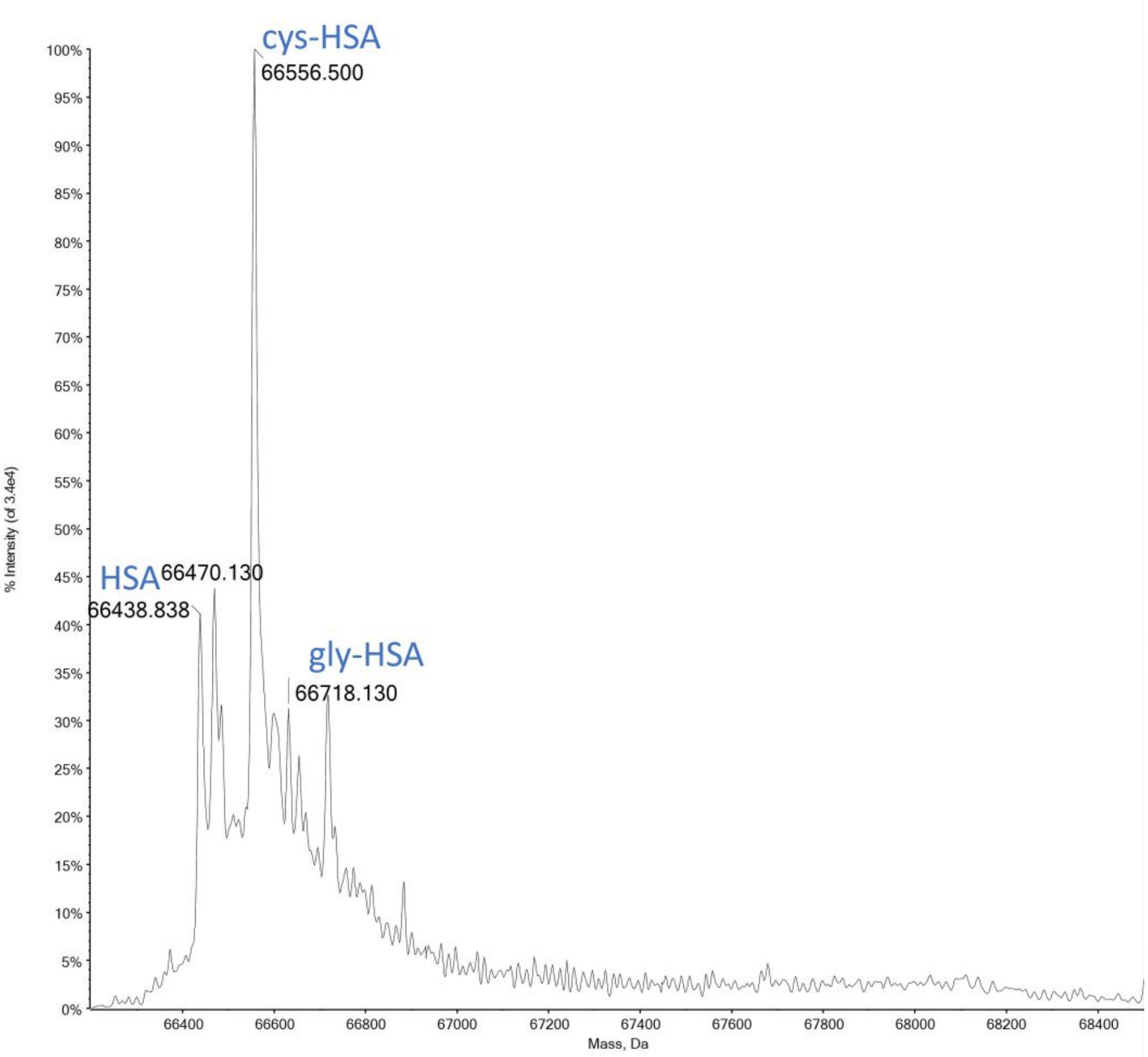

Figure S18. Deconvoluted ESI-Q-TOF spectra of HSA and complex **4** in a 1:1 protein to gold complex ratio at 24 hours.

Emission of compound 5 in the FACS channels

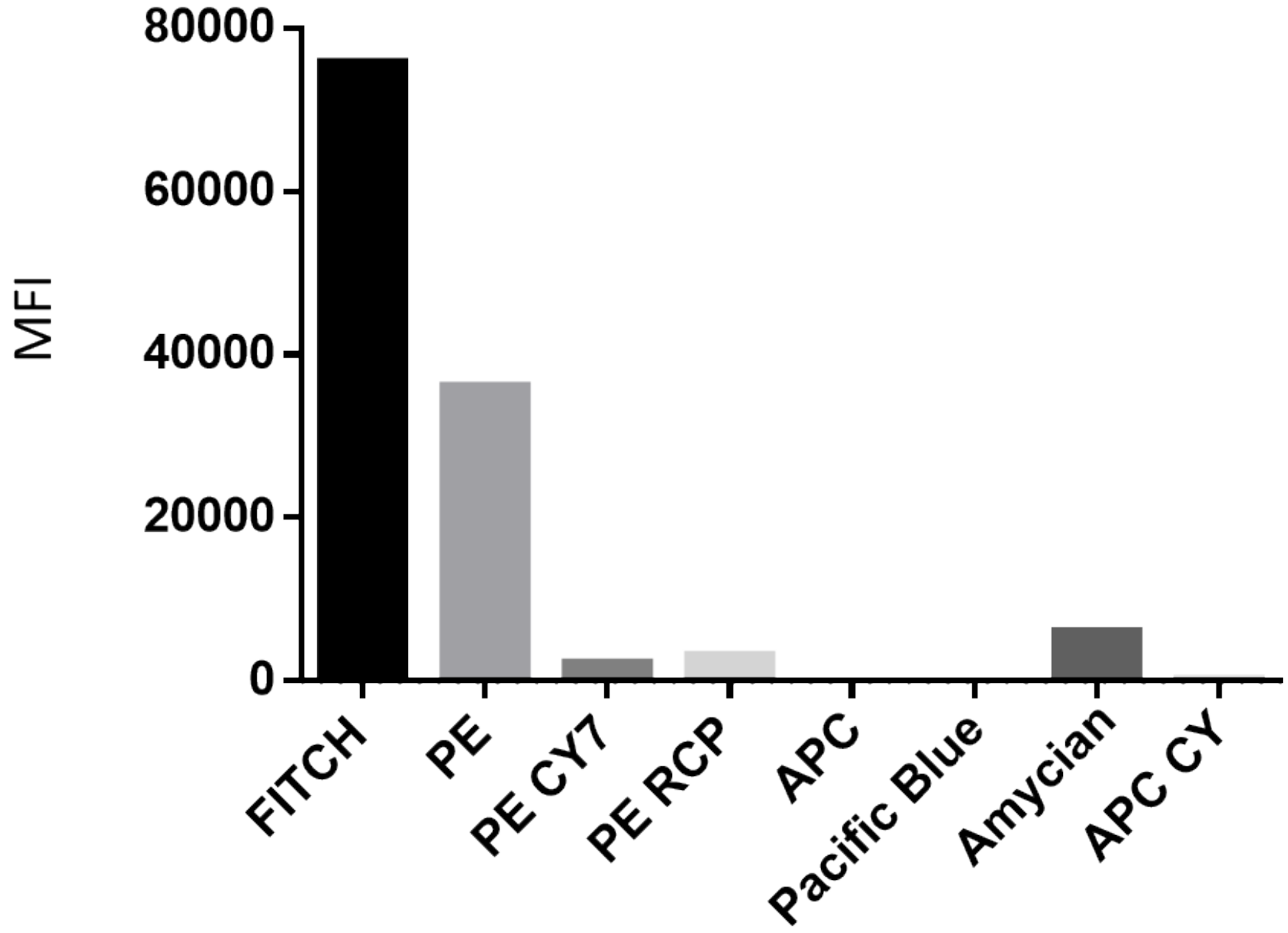

Figure S19. Emission of compound **5** in the FACS channels. Particularly, **5** emits in almost all channels except Pacific Blue, making it challenging to use in biodistribution studies via FACS.
